# Off-line synthesis of evolutionarily stable normative systems

**DOI:** 10.1007/s10458-018-9390-3

**Published:** 2018-06-02

**Authors:** Javier Morales, Michael Wooldridge, Juan A. Rodríguez-Aguilar, Maite López-Sánchez

**Affiliations:** 1 Department of Computer Science, University of Oxford, Oxford, UK; 2 Artificial Intelligence Research Institute (IIIA-CSIC), Campus de la UAB, Bellaterra, Spain; 3 Department of Applied Mathematics and Computer Science, Universitat de Barcelona, Barcelona, Spain

**Keywords:** Norms, Normative systems, Norm synthesis, Evolutionary algorithm

## Abstract

Within the area of multi-agent systems, normative systems are a widely used framework for the coordination of interdependent activities. A crucial problem associated with normative systems is that of synthesising norms that will effectively accomplish a coordination task and that the agents will comply with. Many works in the literature focus on the on-line synthesis of a single, *evolutionarily stable norm* (convention) whose compliance forms a rational choice for the agents and that effectively coordinates them in *one* particular coordination situation that needs to be identified and modelled as a game in advance. In this work, we introduce a framework for the automatic off-line synthesis of *evolutionarily stable normative systems* that coordinate the agents in multiple *interdependent coordination situations* that cannot be easily identified in advance nor resolved separately. Our framework roots in evolutionary game theory. It considers multi-agent systems in which the potential conflict situations can be automatically enumerated by employing MAS simulations along with basic domain information. Our framework simulates an evolutionary process whereby successful norms prosper and spread within the agent population, while unsuccessful norms are discarded. The outputs of such a natural selection process are sets of *codependent norms* that, together, effectively coordinate the agents in multiple interdependent situations and are evolutionarily stable. We empirically show the effectiveness of our approach through empirical evaluation in a simulated traffic domain.

## Introduction

Within human societies and multi-agent systems (MAS), normative systems (norms) have been widely studied as a mechanism for coordinating the interplay between autonomous agents [7, 28]. Norms can resolve coordination problems in MAS by guiding the decision-making of the agents, restricting their behaviours or encouraging desirable courses of action once some preconditions are fulfilled. Coordination in this sense is usually understood as ensuring that the agents can successfully interact by avoiding undesirable outcomes.

When designing norms for MAS (e.g., an autonomous cars scenario), a system designer will potentially need to address two crucial problems.First, identifying all the *conflict situations* (or even the MAS states) in which the agents may require coordination. If performed manually, this task might be time consuming and error prone, specially if we consider that conflict situations might be *interdependent* – that is, the agents’ decisions in a situation might affect the outcomes of different ones. As an example, two cars arriving at a junction will need to decide which one yields in order to avoid collisions. However, the coordination success of these cars might depend on the decisions of other cars in the road, such as other cars queueing behind them (and so on an so forth). These interdependencies might lead to numerous situations that cannot be easily identified in advance nor resolved separately. Secondly, a system designer will need to synthesise an *effective* normative system (a set of norms) that the agents *will comply with* and that will successfully coordinate the agents in all the identified conflict situations. Such a norm synthesis problem is known to be a challenging problem (NP-Complete [30]) that remains open.

A popular (on-line) norm synthesis approach in the literature is that of *norm emergence* (or convention emergence) [3, 5, 26, 27, 31, 33, 36], in which the norms of a MAS emerge from within the agent society at runtime. For the purposes of this paper, what is interesting about norm emergence is that it has been successfully used to synthesise *stable norms*. Most works on norm emergence build on principles in line with those from the framework of *evolutionary game theory* (EGT) [32]. They consider a MAS in which the agents repeatedly engage in a conflict situation modelled as a game. Strategies that are seen to be successful prosper and spread within the agent society through an evolutionary process whereby the agents tend to adopt successful strategies with higher probabilities than unsuccessful ones. A conventional norm (a convention) [36] emerges once a significant proportion of agents adopt a strategy that everyone prefers to conform, on the assumption that everyone else does. Such a strategy is said to be *evolutionarily stable* (ESS), for no agent could benefit from deviating. Hence, complying with an ESS forms a rational choice for the agents.

From the point of view of a system designer, EGT and norm emergence can be seen as powerful tools to synthesise *effective* and *stable* norms. By simulating the evolution of strategies in a MAS, one can anticipate the norms that the agents will abide by at runtime because they lead to successful coordination [3, 31, 34]. However, norm emergence considers a *single* (typically two-player) game that is *known beforehand* and of which the agents have complete information (e.g., the number of players, and the payoffs). Then, *one* norm is synthesised to coordinate the agents in the game. Therefore, norm emergence is inappropriate to synthesise norms for MAS with numerous interdependent conflict situations, such as the traffic scenario. One possible solution might be considering a big game involving all the agents from interdependent conflict situations (e.g., a game with two cars in a junction and other cars queueing behind them). Nevertheless, agents may have a *limited perception* of the environment, thus being unable to properly detect big games that they are engaged in in order to coordinate with all their players. For instance, cars in a junction may not be able to perceive other cars behind them, thus being unaware of their need for coordination.

An alternative on-line norm synthesis approach that may be more appropriate for the scenarios pictured so far is the one proposed in [17, 18], in which the potential conflict situations of a MAS need not be known in advance. Complex coordination situations are detected at runtime during MAS executions by considering basic domain information, and then modelled as sets of smaller, interdependent games that the agents can fully recognise and coordinate in. Norms are automatically created to resolve each game, and individually evaluated in terms of their joint performance once the agents simultaneously play interdependent games. The outputs of this process are sets of norms whose coordination utility depend on each other and, *as a whole*, effectively coordinate the agents in each possible game. However, unlike most state-of-the-art approaches, this work *does not consider evolutionary stability* as a synthesis criterion, and hence the normative systems it synthesises cannot be guaranteed to be evolutionarily stable.

Against this background, this paper builds on techniques from the work in [17, 18] and the framework of EGT [32], and contributes to the state of the art by introducing a novel framework for the *off-line synthesis of evolutionarily stable normative systems* (ESNS) for MAS. Our framework considers a MAS with multiple, interdependent conflict situations that are unknown beforehand. It employs techniques described in [17, 18] to automatically discover these situations during MAS simulations, modelling them as interdependent games. Norms are automatically created in order to coordinate the agents in these games, and submitted to an evolutionary process inspired in EGT. Norms that are proven to be useful to coordinate the agents in each game prosper and spread, ultimately converging to sets of *codependent conventional norms* that, together, are effective for coordination and evolutionarily stable. We provide empirical evaluation of our framework in a simulated traffic domain. We show that it can synthesise ESNSs that successfully avoid conflicts in numerous (up to 89) interdependent traffic situations that cannot be easily identified and resolved separately, for the resulting normative systems would not be evolutionarily stable.

Broadly speaking, our framework provides a valuable tool for off-line norms design. Given a MAS, it opens the possibility of synthesising evolutionarily stable normative systems without requiring full knowledge about its possible conflict situations. By simulating basic agents’ interactions and providing basic domain information (such as identifying when cars collide), our framework can provide system designers with the necessary norms to achieve effective and stable agent coordination in a MAS.

The remainder of the paper is organised as follows. Section 2 provides the necessary background to understand our work. Section 3 describes our framework, whereas Sect. 4 illustrates its empirical evaluation. Section 5 reviews the state of the art in norm synthesis, and Sect. 6 provides some concluding remarks and outlines possible future research. Finally, Sect. 7 discusses the main limiting assumptions of this work and how these might be lifted in order to be applicable to a wider extent of problems.

## Background

In this section we provide the necessary background to understand our work. We start by surveying the automatic norm synthesis approach described in [17, 18], named iron (*Intelligent Robust On-line Norm Synthesis*), as our work employs it to automatically detect coordination situations and generate norms. Then, we introduce the framework of evolutionary game theory (EGT) [32] and the key concept of *evolutionarily stable strategy* (ESS).

### Automatic norm synthesis

iron is an iterative approach that monitors the evolution of a MAS at regular time intervals, searching for *conflict situations* (e.g., collisions in a traffic scenario). Whenever it detects a conflict at time *t*, iron triggers a *norm generation* process that results in the creation of a norm aimed to avoid the detected conflict in the future (from time $t+1$ onwards). The norm is then communicated to the agents, and thereafter empirically evaluated in terms of its *utility* to coordinate the agents once they comply with it.^1^ If the norm avoids conflicts once the agents abide by its strictures, then it is regarded as useful and sustained over time. Otherwise, the norm is ultimately discarded and alternative norms are created in order to avoid the conflict. Over time, iron may detect multiple, interdependent conflicts. As a result, it will create multiple, codependent norms whose utility might depend on each other.

**Fig. 1:**
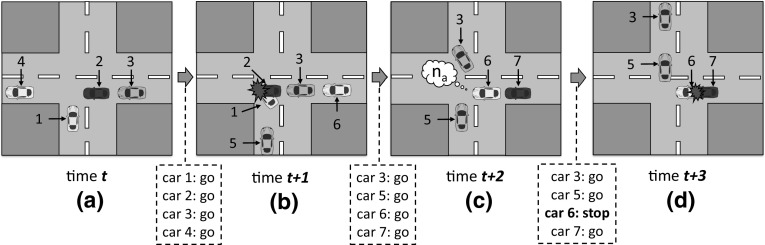
Evolution of a traffic junction over time: **a** At time *t* there are four cars in the junction. Cars 1, 2 and 3 require coordination in order to avoid collisions; **b** at time $t+1$, all cars have performed action *“go”*. As a result, cars 1 and 2 collide; **c** At time $t+2$, car 3 goes forward (it also turns right), and cars 5 and 6 engage in the same conflict situation played by cars 1 and 2 at time *t*. This time, car 6 has applicable norm $n_a$ (*“give way to your left”*), which prohibits it to go forward. Also, car 7 needs to coordinate with car 6 in order to not collide, but it has no norms for this aim; **d** At time $t+3$, car 5 avoids colliding thanks to car 6 yielding, but car 6 is hit by car 7 from behind

For the purposes of this paper, we are interested in how iron detects conflicts and generates norms. For clarity, we will rely on a running example to detail iron’s norm generation. Let us consider a traffic scenario where agents are cars, and the coordination task is to ensure that cars interact without colliding. The actions available to the cars are *“go”* forward^2^ and *“stop”*. Figure 1a depicts a state of this scenario at time *t*, with four cars. Of these, cars 1–3 are engaged in a conflicting situation. If cars 1 and 2 go forward, they will collide. If car 2 stops, giving way to car 1, then car 3 has to stop in order to avoid hitting car 2 from behind. Alternatively, car 1 can stop, giving way to cars 2 and 3.

iron considers a MAS in which the agents have a *limited perception* of the environment. Thus, a car perceives the road by means of its *local context*, i.e., its internal representation of the area of the road that is immediately next to and in front of it at a given time. For instance, cars 1 and 2 perceive each other at time *t* (Fig. 1a), but neither of them can perceive car 3. Car 3 perceives car 2, but it cannot perceive car 1.

iron detects a conflict at a given time by perceiving the state of the monitored MAS, and employing a domain-dependent *conflict function*^3^ that allows it to detect groups of agents whose interaction has led to an undesirable outcome in the state. For instance, at time $t+1$ (Fig. 1b), iron will detect a collision between cars 1 and 2 after both have performed action *“go”*.

After detecting a collision at time $t+1$, iron creates a norm aimed to *avoid future collisions*. Specifically, it creates a norm that prohibits to perform one of the actions performed by the collided cars at time $t+1$ whenever they engage in the situation faced by them before colliding, i.e., at time *t*. In this way, the conflict might be avoided in the future. iron creates a norm as follows. First, it randomly chooses one of the cars involved in the collision at time $t+1$, e.g., car 2. Then, it employs a domain-dependent ${context function}^3$ to retrieve the local context of this car in the situation previous to the conflict. The context of car 2 at time *t* can be informally described as *“there is a car coming from my left”*.^4^ Next, it employs a domain-dependent ${action function}^3$ to retrieve the *action* that this car performed during the transition from time *t* to time $t+1$ (i.e., action *“go”*).

Finally, iron creates a norm that the agents can interpret and comply with. Such a norm will have the retrieved car’s context as its activation condition (precondition), and a *prohibition* to perform action *“go”* as its postcondition. Thus, by using this norm, any car perceiving a car coming from its left will be prohibited to *“go”* forward. The resulting norm, $n_a$, can be informally described as:

$n_a$: **if** *there is a car coming from my left* **then** *“i am prohibited to go”*.

The new norm is then communicated to all the agents in the system, who will have this norm applicable once they face the situation described by the norm. Then, iron keeps monitoring the MAS. At time $t+2$, cars 5 and 6 engage in the same situation faced by cars 1 and 2 at time *t*. This time, car 6 has norm $n_a$ applicable and is prohibited to go forward. Consequently, car 6 stops at time $t+3$, giving way to car 5, and being hit by car 7 from behind. iron will detect this new conflict, which in fact is caused by the decision of car 6. Then, it will generate a new norm that prohibits to go to any agent encountering the context of car 7 at time $t+2$. The resulting norm, $n_b$, can be described as:

$n_b$: **if** *there is a car in front of me going forward* **then** *“i am prohibited to go”*.

which can be seen as a “security distance” norm.

Notice that the two norms synthesised so far ($n_a$ and $n_b$) are *codependent*, i.e., they can only avoid collisions if the agents have *both* of them. iron has no means to explicitly detect such a codependency relationship. Instead, it implicitly captures norms’ codependencies effects by empirically computing their individual utilities in a series of concurrent game plays in which norms can affect each other’s performances. For instance, say that a car applies norm $n_a$ and the car behind it applies norm $n_b$, hence avoiding collisions. Then, both norms will be individually evaluated as useful. Conversely, should cars not have and apply $n_b$, then norm $n_a$ would be individually evaluated as useless. Eventually, iron will sustain these norms if they individually prove to be useful enough during a sufficient amount of time. Otherwise, it will discard them and will synthesise new ones, and so on and so forth until it finds a set of norms that successfully avoid conflicts in all possible situations.

At this point it is worth to notice that the normative systems synthesised by iron **cannot be guaranteed to be evolutionarily stable**. iron does not seek to synthesise *optimal* normative systems, but normative systems that are *good enough* for a given MAS. Thus, once iron finds a good enough normative system, it misses to explore “better” (more useful) normative systems that the agents might eventually be tempted to switch to. In this work we address this problem, building on iron and incorporating ideas from EGT in order to synthesise evolutionarily stable normative systems.

### Evolutionary game theory

EGT combines population ecology with classical game theory. It considers a population of agents that repeatedly engage in strategic pairwise interactions by adopting different (pure) strategies. An ESS is a strategy that, if adopted by a majority of agents, no agent could benefit from using any alternative strategy – namely, the *fitness* (i.e., the average payoff) of an agent using that strategy is higher than the fitness of any agent using alternative strategies.

EGT provides a model of the underlying process whereby strategies change in a population. It assumes that successful strategies “reproduce”, growing in frequency with higher probabilities than less successful strategies. Evolutionarily stable strategies are attractor points of such a natural selection process whereby agents can converge to adopting an ESS.

**Fig. 2:**
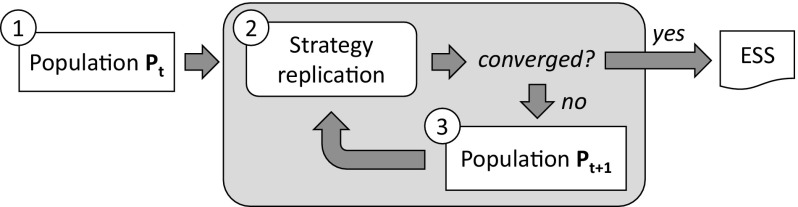
EGT model, composed of three phases: (1) a population $P_t$ is created in which each group of strategists has a certain size; (2) each group of strategists grows in terms of their fitness when playing against different strategists; (3) if the process converges, it returns an ESS. Otherwise, it replicates a new population $P_{t+1}$ in which each group of strategists has grown in numbers proportional to its fitness

Figure 2 graphically illustrates the EGT model. It considers an initial population of agents $P_t$ that adopt different strategies to play a game (Fig. 2(1)). Each strategy has a certain *fitness* that quantifies the average payoff to an agent that adopts the strategy to play against other strategists. Strategies are then replicated (Fig. 2(2)), growing in frequency proportionally to their relative fitness with respect to the average fitness of the population. If the process has not converged yet, then a new population $P_{t+1}$ is generated that reflects the changes in strategy frequencies (Fig. 2(3)). Such population is then employed to repeat the replication process, and so on and so forth until the process converges. The replication process is considered to have converged once the population remains stable between generations (that is, the frequencies of each strategy remain unchanged). Then, if a majority of agents have adopted the same strategy, this strategy is considered to be an ESS.

Next, we detail the equations employed by EGT to perform strategy replication, also known as the *replicator dynamics*. We illustrate the replicator dynamics by relying on the game of the Prisoner’s Dilemma [20], illustrated in Table 1. In this game, two agents can choose either to cooperate (C) or to defect (D) in some situation. If both agents cooperate, both receive a *reward* payoff 3. If both defect, both receive a *punishment* payoff 2. If one agent defects and the other cooperates, the defector receives a *temptation* payoff 5, while the cooperator receives the *sucker’s* payoff, 1.

**Table 1:** Payoff matrix of the Prisoner’s Dilemma

	**C**	**D**
**C**	(3,3)	(0,5)
**D**	(5,0)	(1,1)

Two agents can choose to cooperate (C) or to defect (D). If both agents cooperate, both receive a *reward* payoff 3. If both defect, both receive a *punishment* payoff 1. If one agent defects and the other cooperates, the defector receives a *temptation* payoff 5, while the cooperator receives the *sucker’s* payoff, 0

Consider a population of agents that adopt either strategy C or strategy D. Let $F( C ) \in [0,1]$ be the frequency of cooperators in the population, and $F( D ) \in [0,1]$ the frequency of defectors. Note that $F( C ) + F( D ) = 1$. Let us denote as $\rho ( C,D )$ the payoff to a cooperator when playing against a defector, and analogously for other strategy pairs. We assume that each strategist has an initial fitness $f_0$. The fitness of each strategy will depend on: (1) the **payoff** to an agent when interacting with either a cooperator or a defector, and (2) the **probability** to encounter each one of these, which actually is a representation of the frequency of strategists of each type. The fitness *f* of each strategy can be computed as:

1 $$\begin{aligned} f( C )= & {} f_0( C ) + F( C ) \cdot \rho ( C,C ) + F( D ) \cdot \rho ( C,D ) \end{aligned}$$

2 $$\begin{aligned} f( D )= & {} f_0( D ) + F( C ) \cdot \rho ( D,C ) + F( D ) \cdot \rho ( D,D ) \end{aligned}$$

$f_0(C)$ and $f_0( D )$ being the initial fitness of cooperators and defectors, respectively.

In this manner, the fitness of a cooperator is computed as the summation of its initial fitness, the probability of encountering a cooperator times the payoff to the cooperator when that happens, and the probability of encountering a defector times the payoff to the cooperator when that happens. The fitness of a defector is computed analogously.

Agents reproduce in numbers proportional to their fitnesses. In next generation, the frequency of cooperators and defectors is updated in terms of their relative fitnesses with respect to the average fitness of the whole population. Then, if cooperators perform worse than average they will decrease their frequency, and if defectors perform better than average they will grow in frequency. The frequencies of cooperators and defectors are updated as: Replication equation changed to an alternative one used in the literature. This one can be used if norms’ fitnesses are guaranteed to be always positive.

3 $$\begin{aligned} F( C )' = F( C ) \cdot f( C ) \ / \ \theta \end{aligned}$$

4 $$\begin{aligned} F( D )' = F( D ) \cdot f( D ) \ / \ \theta \end{aligned}$$

where $\theta$ is the weighted average fitness of the whole population, computed as:

5 $$\begin{aligned} \theta = F( C ) \cdot f( C ) + F( D ) \cdot f( D ) \end{aligned}$$

In biology, replication models the natural process whereby fittest individuals are more likely to survive and to reproduce than less fit ones. In economic settings (such as multi-agent systems), replication provides a model of imitation [6, 12] whereby the agents tend to imitate strategists that prove to perform well, i.e., have a higher fitness, thereby adopting their strategies over time. Then, if a strategy is fitter than the average, agents will be more likely to adopt it than to adopt a less fit one.

As previously mentioned, the replication process can eventually lead the population to a point of equilibrium in which the frequencies of each strategy do not change over time because their fitnesses are equal. When this happens, the population can be either *monomorphic* (a majority of agents adopt the same strategy) or *polymorphic* (the agents adopt a variety of strategies). If the population composition can be restored after a disturbance,^5^ then it is said that the population is in an *evolutionarily stable state*. If such population is monomorphic, then the strategy adopted by the agents is said to be an ESS.

In the Prisoner’s Dilemma, cooperation is not an ESS because a population of cooperators will always perform more poorly (will have a lower fitness) than defectors when playing against each other. Then, defection is the only evolutionarily strategy: a population of cooperators can be invaded by one defector.

## Evolutionary norm synthesis

In this section we introduce our framework for the synthesis of *evolutionarily stable normative systems* (ESNS) – hereafter, referred to as our “System for Evolutionary Norm SynthEsis”, or sense for short. In Sect. 3.1, we provide a general overview of the sense operation. Then, we provide some basic definitions and formally define our problem in Sect. 3.2. We detail how sense performs evolutionary norm synthesis in Sect. 3.3.

### Evolutionary norm synthesis system: an outline

Our framework implements an evolutionary process similar to the one in EGT (Sect. 2.2), but instead of strategies, it replicates norms. It considers a MAS in which agents have a *limited perception* of the environment. Thus, complex coordination situations of a MAS, in which agents may not be able to entirely perceive each other in order to detect their need for coordination, are resolved by creating sets of simpler, *interdependent games* in which agents can fully perceive each other and coordinate. At a given time, the coordination success of the agents playing a game will depend on their actions in the game, as well as the actions of other agents simultaneously playing interdependent games.

sense *assumes no previous knowledge* about the potential games of a MAS neither about the potential payoff structure of the MAS. It *learns* these games along with their payoffs from the observation of agents’ interactions within *MAS simulations*, and automatically creates norms to coordinate the agents in them. For each game, sense endows each one of its norms with: (i) a *fitness* value that quantifies its average utility to coordinate the agents in the game; and (ii) a *frequency* value that stands for the proportion of agents that *have* the norm. These norms will *compete with each other* for survival. Norms that prove to be fitter in a game will prosper and will increase their frequency, while the less fit ones will be ultimately discarded. Over generations, only the fittest norms of each game will survive, achieving 100% frequency. Since games are interdependent, their respective norms will be *codependent* and hence they will *co-evolve* over time. Eventually, norm co-evolution can converge to a set of *conventional norms* (one for each game) that, *together*, achieve coordination in each possible game and are evolutionarily stable.

**Fig. 3:**
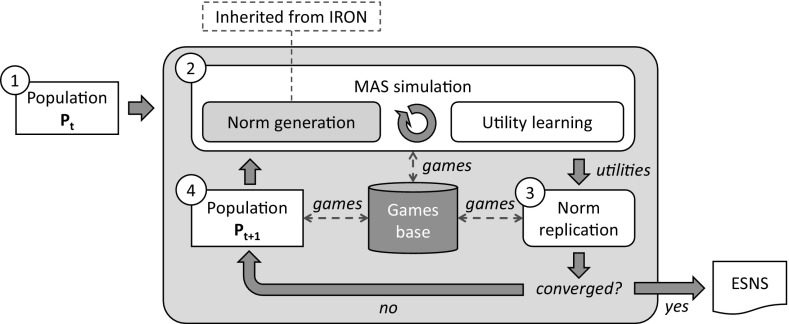
Graphical representation of sense. It starts in (1), with an initial agent population $P_t$; (2) simulation is performed, during which sense detects new games and creates norms, and empirically computes norms’ utilities; (3) norms are replicated, growing in numbers proportional to their fitness; (4) a new population $P_{t+1}$ is generated in which the size of the group of agents adopting each norm is proportional to the frequency of the norm. Such new population is employed in a new iteration of the process

Figure 3 illustrates the process implemented by sense. Likewise EGT, it starts with an initial population $P_t$ (Fig. 3(1)) whose agents employ no norms to coordinate. Each agent in $P_t$ has a personal *normative system* (a set of norms, one for each possible game) that is initially empty. sense proceeds by repeatedly performing the following tasks:

- **MAS simulation** sense runs simulation of the MAS for a certain amount of time,^6^ repeatedly carrying out two simultaneous subtasks:
  - ***Norm generation***, which consists in monitoring agents’ activities, detecting new games and keeping track of them in a *Games Base*. For each new tracked game, sense creates alternative norms to coordinate the agents and sends each norm to different agents. For instance, if a game has four different norms, 25% of agents will be provided with the first norm, and the same applies to the remaining norms. The agents will incorporate their assigned norms to their normative systems, and thereafter, every time they play the game, their respective norms will guide their courses of action for the sake of coordination. Over time, sense will create a heterogeneous population whose agents have different normative systems, and thus will play each game by using different, *competing* norms. In order to detect conflicts and create norms, sense employs iron’s techniques described in Sect. 2.1.
  - ***Utility learning***, which consists in accumulating evidence about the *utilities* of the norms of each game once the agents play the game over time. A norm’s utility is ***empirically*** computed in terms of the frequency with which it effectively coordinates the agents in a game once they comply with the norm within a sequence of game plays. Such a utility might depend on the (codependent) norms adopted by the agents in simultaneously played interdependent games. Computing norms’ utilities empirically allows sense to capture in a compact manner the runtime effects of the codependencies between norms, enabling it to evaluate and co-evolve norms *together*.
- **Norm replication** After simulation, sense has available a collection of interdependent games along with their norms’ utilities. With all this at hand, sense replicates norms likewise strategies are replicated in EGT. For each game, it computes the *fitnesses* of its competing norms as their average utility in the game. Then, it replicates each norm in numbers proportional to its fitness: the *frequency* of those norms fitter than average will increase, while that of those norms less fit than average will decrease. Likewise utilities, the fitnesses and frequencies of codependent norms will co-evolve over time.

After replication, if the process has not converged yet, a new population $P_{t+1}$ will be generated (Fig. 3(4)) in which the size of the group of agents having each norm will be proportional to the new frequency of the norm. Thus, if a norm is 50% frequent, then 50% of agents will *have* the norm. This new population will be then employed for a new simulation and replication, and so on and so forth until either the process converges, or it reaches a time out$^6$. We say that sense has converged once the following conditions hold:

1. There is *one* norm in each game that has eliminated its competitors, i.e., this norm has achieved 100% frequency.
2. The frequencies of each norm of each game remain stable (unchanged) during a sufficient amount of generations, denoted as ${\mathcal {I}}$.

Once this happens, the agent population has converged to using the same norms in a set of games. Then, we say that the agents have adopted an *evolutionarily stable normative system*.

**Fig. 4:**
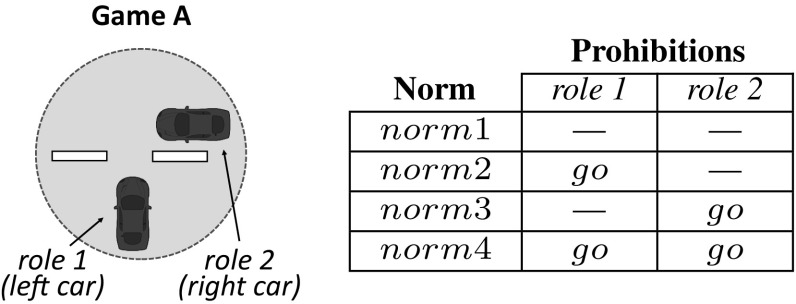
Left: game where two cars with roles 1 and 2 need coordination when approaching a junction. Right: alternative norms to coordinate any two cars that engage in such a game

**Fig. 5:**
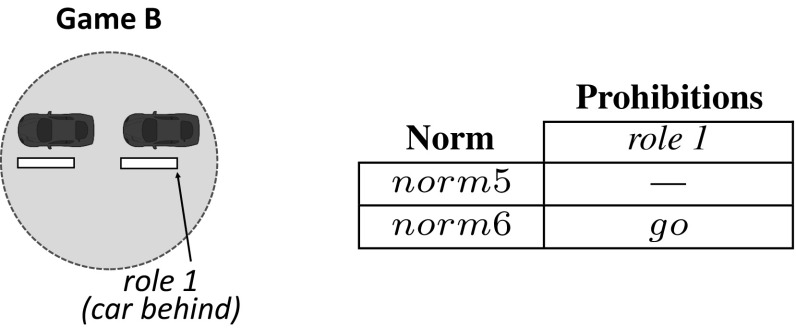
Left: game where a car behind (role 1) has to coordinate with a car in front in order to not collide. Right: alternative norms to coordinate any car that engages in such a game

Let us illustrate the operation of sense with our traffic example introduced in Sect. 2.1. Consider an initial car population with no norms to coordinate. Cars have a *limited perception of the MAS*, and can perceive the area of the road immediately next to and in front of them (they cannot perceive what is behind). Say that, after simulation, sense has detected two interdependent games illustrated in Figs. 4 and 5. Figure 4 depicts a *one-shot* game A in which two cars *perceive one another* in a junction. Thus, this game has two *roles*: a car coming from the left (role 1) and a car coming from the right (role 2). Both roles have available actions *“go”* and *“stop”*. sense will create the following norms, listed in the Table of Fig. 4:

- *norm*1 establishes no prohibitions. Thus, a car is free to go forward when coming from either the left or the right (no matter the role it plays).
- *norm*2 says that a car is prohibited to go forward when coming from the left (when playing role 1). In practice, *norm*2 stands for a “give way to the right” norm. Analogously, *norm*3 stands for a “give way to the left” norm.
- *norm*4 stands for a “give way always” norm, i.e., it prohibits a car to go forward once it is playing either role 1 or role 2.

Similarly, Fig. 5 depicts a game B in which a car behind perceives a car in front, but *not the other way around*. Therefore, this game has a *single role* corresponding to the only car that is aware of the need for coordination of these two cars, i.e., the car behind. This role has two available actions *“go”* and *“stop”*. The norms for this game are illustrated in the Table of Fig. 5: *norm*5 establishes no prohibitions, and *norm*6 prohibits role 1 to go forward.

**Fig. 6:**
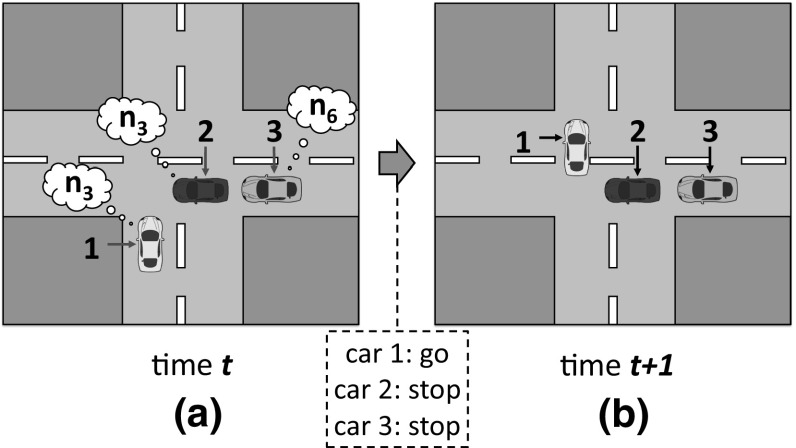
**a** Two cars playing game A from Fig. 4 at time *t*. Both cars coordinate by using *norm*3 (represented as a thought bubble containing $n_3$); **b** successful coordination of the two cars at time $t+1$, after car 2 has given way to car 1

**Fig. 7:**
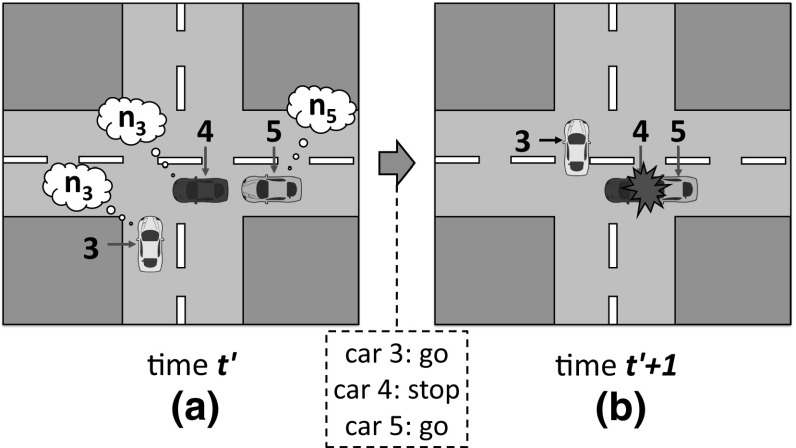
**a** Cars 3–4 playing game A (Fig. 4) at time $t'$ and car 5 playing game B (Fig. 5). Cars 3–4 use *norm*3 to coordinate, and car 5 uses *norm*5; **b** successful coordination of cars 3–4 at time $t'+1$, and unsuccessful coordination of cars 4–5

Each car will incorporate two norms to its normative system: one norm to coordinate in game A, and another norm for game B. In particular, 25% of the cars will have *norm*1 in their normative systems, and the same applies to *norm*2, *norm*3 and *norm*4. Similarly, 50% of the cars will have *norm*5, and the remaining 50% will have *norm*6. Thereafter, sense will accumulate evidence about the coordination utility of each norm once the agents use it to coordinate in a game. For instance, suppose that at a given time *t* sense perceives the situation illustrated in Fig. 6a. Cars 1–2 play game A described above, and car 3 plays game B. Cars 1–2 have *norm*3 as their norm to coordinate in game A, and car 3 has *norm*6 for game B. Thus, at time $t+1$ (Fig. 6b) cars 2 and 3 stop, giving way to car 1 and avoiding a collision. sense will monitor this positive outcome and will evaluate *norm*3 and *norm*6 as useful. At a later time $t'$, sense perceives a similar situation in which car 5 has *norm*5 to coordinate in game B (Fig. 7a). Thus, at time $t'+1$ (Fig. 7b) car 4 stops and car 5 goes forward, hitting it from behind. Consequently, sense will evaluate *norm*3 and *norm*5 as useless.

During norm replication, sense will update the frequencies of *norm*1–*norm*4 for game A. The new frequency of *norm*3 will depend on its relative fitness with respect to the average fitness of *norm*1–*norm*4. As for game B, the frequency of *norm*6 will increase because it has proven to be more useful than *norm*5 (the frequency of *norm*5 will decrease). Then, in next generation sense will create a new agent population $P_{t+1}$ in which a higher number of agents will have *norm*6. Consequently, in next generation *norm*3 will increase its utility because cars will be more likely to stop in game B. Over time, *norm*3 and *norm*6 will co-evolve and their frequencies will jointly increase, possibly leading sense to converge to an ESNS containing these two norms.

### Basic definitions and problem statement

We consider a MAS with a set of agents *Ag* and a finite set of actions *Ac* available to these agents. Let *S* be the set of all the states of the MAS. We adopt a synchronous model in which the agents interact in some system state, perform a collection of actions, and lead the system from its previous state to a new one. We assume that each agent has a limited perception of the state of the MAS it is part of at a given time. Thus, an agent’s *context* stands for its internal representation of a MAS state (i.e., its beliefs). Agents express their contexts in terms of an *agent language* ${\mathcal {L}}$ composed of predicates and terms.

In each state of the MAS, agents may engage in *local* strategic interactions in which they need coordination in order to avoid conflicts. We will refer to such interactions as one-shot *games*. Formally, a game is composed of a set of *roles* that define the *actions* available to each agent involved in the game. A game has a *description* that describes the initial situation of the game from the point of view of its players. Such a description is composed of the local contexts of each player. We assume that the *payoffs* to the players of a game cannot be assessed beforehand, as these might depend on the actions of the players of interdependent games simultaneously played at runtime. Thus, it is important to notice that a game in our model does not contain a predefined payoff matrix. Instead, we define it as follows:

#### Definition 1

(*m*-role game) An *m*-role game is a tuple $\langle R, A, \varphi \rangle$, where:

- $R=\{1, \ldots , m\}$ is a set of *m* agent roles, one per each agent involved in the game.
- $A=\langle A_1, \ldots , A_m \rangle$ is an *m*-tuple of action sets available to each role, where $A_i \subseteq Ac$ is the set of actions available to the agent taking on role *i*.
- $\varphi$ is an expression of ${\mathcal {L}}$ that describes the initial situation of the game from the point of view of its players (i.e., $\varphi$ is the conjunction of their local contexts, sorted by role).

For simplicity, henceforth we shall refer to an *m*-role game as a game, and to an agent playing role *i* in a game as player *i*.

As an example, we can formally describe game A in Fig. 4a as a tuple $G_A=\langle R, A, \varphi \rangle$, where $R=\{1,2\}$ is the set of roles, $A=\langle \{go,stop\},\{go,stop\}\rangle$ is the set of actions available to each role, and $\varphi$ is its description, which can be informally interpreted as *“player 1 perceives a car coming from its right and player 2 perceives a car coming from its left”*.

In general, in each MAS state the same game can be simultaneously played by different groups of agents, and each agent can engage in the same game in different MAS states. At a given time, an agent identifies whether it is engaged in a game in the current state of the MAS (and the role that it plays in it) by means of its context. For instance, if the context of a car at a given time is *“there is a car coming from my right”*, then this car will know that it is enacting role 1 in game $G_A$.

If *G* is a game, a *norm* stands for a coordination strategy that specifies what an agent is *prohibited* to do when playing each possible role of *G*. Norms are expressed in terms of the agent language ${\mathcal {L}}$ so that agents can interpret and comply with them. Formally, a norm is a (possibly empty) set of constraints that restricts the action space of the agents involved in a game by prohibiting certain actions.

#### Definition 2

(Norm) Given a game $G=\langle R, A, \varphi \rangle$, a norm to coordinate the agents in *G* is a pair $\langle \psi , prh \rangle$ where:

- $\psi \in {\mathcal {L}}$ is the precondition of the norm.
- $prh: R \rightarrow 2^{Ac}$ is a function that returns the set of actions that an agent is prohibited to perform when taking on role *i*, where $prh(i) \in 2^{A_i}$ for all $i \in R$.

As an example, *norm*3 introduced above to coordinate cars in $G_A$ can be formally defined as a pair $norm3 =\langle \psi , prh \rangle$, where $\psi =$*“player 1 perceives a car coming from its right and player 2 perceives a car coming from its left”*, and function *prh* returns an empty set for role 1, and action *“go”* for role 2. Formally, $prh(1)=\emptyset$ and $prh(2)=\{go\}$.

Let $G=\langle R, A, \varphi \rangle$ be an *m*-role game, and $n = \langle \psi , prh \rangle$ a norm. We say that *n* applies in *G* if the precondition of *n* satisfies the description of *G*, namely if $\varphi \models \psi$. Hereafter, we will refer to the set of norms that apply in a game *G* as the *norm space* of game *G*, denoted by $N_{G}$. For instance, the norm space of game $G_A$ can be denoted by $N_{G_A}=\{norm1, norm2, norm3, norm4\}$ (the norms in the Table in Fig. 4).

Agents in a MAS may engage in multiple, different games. Henceforth, we shall denote the set of games that agents can play as ${\mathcal {G}} = \{G_1,\ldots ,G_s\}$. A normative system is a set of $|{\mathcal {G}}|$ norms that provides an agent with the means to coordinate in each game in ${\mathcal {G}}$. Following our example, each car will have one norm out of norm space $N_{G_A}$, and so on for each game.

#### Definition 3

(Normative system) Let ${\mathcal {G}}$ be a (possibly empty) set of games. A normative system is a set of norms $\varOmega$ such that for each $G \in {\mathcal {G}}$ there is one norm $n \in \varOmega$ and $n \in N_{G}$.

First of all, each agent $ag_j \in Ag$ counts on its own normative system $\varOmega _j$. Thus, in general we assume that a MAS is composed of a heterogeneous population whose agents may have different normative systems.

Let $Ag' \subseteq Ag$ be a group of agents engaged in a game $G=\langle R, A, \varphi \rangle$ at a given time, each playing one role from *R*. Each agent will count on one norm out of its normative system that applies in *G* and prohibits it to perform some actions. We denote the combination of norms applicable to these agents at this given time as $\mathbf {n}=\langle n_1, \ldots , n_m \rangle$, where $n_i$ stands for the norm for *G* in the normative system of the agent playing role *i*. We assume that agents *always comply* with their applicable norms.^7^ Therefore, based on the norms in $\mathbf {n}$, these agents will perform a tuple of actions $\mathbf {a}=\langle a_1,\ldots ,a_m \rangle$, where $a_i$ is an action performed by the agent enacting role *i* that is *not prohibited* by norm $n_i$ for role *i*.^8^

As previously introduced, we assume that the *payoffs* to the players of a game cannot be assessed beforehand, as these might depend on the actions of the players of interdependent games simultaneously played at runtime. However, we can compute the *rewards* to the players of such a game *at a given time*, once they perform a joint action and lead the MAS to its next state. We obtain such a reward by means of a *reward function*.

#### Definition 4

(Reward) Given a MAS with a set of agents *Ag*, the reward to an agent $ag \in Ag$ at a particular point in time $t \in {\mathbb {N}}$ is represented by means of a reward function of the form $r^t(ag) \in [0,1]$.

Say that cars 1 and 2 in Fig. 6a have normative systems $\varOmega _1$ and $\varOmega _2$, respectively, and that both normative systems have *norm*3 as the applicable norm in $G_A$. Thus, at time *t* these cars play $G_A$ with norm combination $\mathbf {n}=\langle norm3, norm3 \rangle$. These cars will perform a joint action $\mathbf {a}=\langle go,stop \rangle$, thus avoiding collisions at time $t+1$ and getting reward 1 (i.e., $r^{t+1}(1)= 1$ and $r^{t+1}(2)= 1$). At time $t'$ (Fig. 7a), cars 3 and 4 play $G_A$ with the same norm combination. Car 3 avoids collisions, but car 4 is hit from behind by car 5, which was engaged in game B. Thus, the rewards of cars 3 and 4 at time $t'+1$ are 1 and 0, respectively, i.e., $r^{t'+1}(3)= 1$ and $r^{t'+1}(4)= 0$.

Notice that, in practice, given a game and the norms that apply in it, the agents will play a repeated one-shot game of norms against norms, namely a *normative game*, in which the norm combinations used by the agents to play the game over time will lead them to obtain a *history* of rewards. Thus, a normative game will consist of a game, the norms that apply in it, and a history of rewards obtained by the agents in the game.

#### Definition 5

(Normative game) A normative game is a tuple $\langle G, N_{G}, H, U \rangle$, where:

- *G* is an *m*-role game, and $N_{G}$ is the norm space of *G*.
- $H=\langle h_0,\ldots , h_w \rangle$ is the memory of the normative game over a time window $[0,t_w]$, where $h_j=\langle \mathbf {n}^j, \mathbf {r}^j \rangle , j \in [0,w]$ such that $\mathbf {n}^j \in N_G^{|R|}$ is the combination of norms that applied to the agents playing the game at time $t_j$, and $\mathbf {r}^j$ is the vector of rewards that these agents obtained at that time (one for each agent).
- $U=\langle u_1, \ldots , u_m \rangle$ is an *m*-tuple of utility functions of the form $u_i: N_G^{|R|} \times H \rightarrow [0,1]$, which return the personal coordination utility to an agent enacting role *i* in the game once the players of the game have a certain combination of applicable norms. Such a utility is empirically computed based on the memory of the game, *H*.

Intuitively, the utility of a norm combination tells us how successful it has been historically to avoid conflicts to each player of the game. Such a utility is computed based on the rewards obtained by the agents that have played the game within a time window. Further on, we provide equations to compute such a utility in Sect. 3.3.2.

Note therefore that each game will have its equivalent normative game. If ${\mathcal {G}}=\{G_1, \ldots , G_s \}$ is a set of games with norm spaces $N_{G_1}, \ldots , N_{G_s}$, we shall denote as ${{\mathcal {N}}}{{\mathcal {G}}}=\{NG_1, \ldots , NG_s\}$ the set of all possible normative games, where $NG_i$ is the normative game resulting from $G_i$ and the norms from $N_{G_i}$.

At this point we import from EGT the concept of *fitness* introduced in Sect. 2.2. Given a normative game *NG*, the fitness of each one of its norms quantifies the *average utility* of an agent that uses the norm to play *NG* by enacting different roles and by playing against agents with different norms. Formally:

#### Definition 6

(Norm fitness) Given a normative game $NG=\langle G, N_{G}, H, U\rangle$, the fitness of a norm $n \in N_{G}$ is represented by means of a function of the form $f(n,NG) \in [0,1]$.

Furthermore, given a collection of normative games ${{\mathcal {N}}}{{\mathcal {G}}}$, and a normative system $\varOmega$ with one norm for each normative game $NG \in {{\mathcal {N}}}{{\mathcal {G}}}$, we compute the *global fitness* of normative system $\varOmega$, denoted by $f_{{\mathcal {G}}}$, as the aggregated fitness of the norms of $\varOmega$ in their respective normative games:

6 $$\begin{aligned} f_{{\mathcal {G}}}(\varOmega , {{\mathcal {N}}}{{\mathcal {G}}}) = \sum \limits _{NG \in {{\mathcal {N}}}{{\mathcal {G}}}} f(n^{\varOmega }_{NG},NG) \end{aligned}$$

where $n^{\varOmega }_{NG}$ stands for the norm from $\varOmega$ to coordinate the agents in normative game *NG*.

Now we are ready to introduce the problem that we address in this paper. Consider a population of agents, and a collection of interdependent normative games ${{\mathcal {N}}}{{\mathcal {G}}}$. Our aim is to find a normative system $\varOmega$ such that, once it is used by all the agents to coordinate in the games in ${{\mathcal {N}}}{{\mathcal {G}}}$, there is no agent that can derive a greater global fitness by using an alternative normative system $\varOmega '$. In terms of EGT, this amounts to saying that normative system $\varOmega$ is *evolutionarily stable*, since no agent could be ever tempted to use alternative normative systems to coordinate in the normative games in ${{\mathcal {N}}}{{\mathcal {G}}}$.

#### Definition 7

(Norm synthesis problem) Given a set of agents *Ag* and a set of normative games ${{\mathcal {N}}}{{\mathcal {G}}}$, our aim is to find a normative system $\varOmega$ such that the following conditions hold:

1. **All agents adopt** $\varOmega$. That is, $\varOmega _i = \varOmega$ for each agent $ag_i \in Ag$.
2. **There is no alternative normative system whose fitness outperforms that of** $\varOmega$. Formally, there is no alternative normative system $\varOmega '$ such that $f_{{\mathcal {G}}}(\varOmega ',{{\mathcal {N}}}{{\mathcal {G}}}) > f_{{\mathcal {G}}}(\varOmega ,{{\mathcal {N}}}{{\mathcal {G}}})$.

### Formal model for evolutionary norm synthesis

We now describe the tasks that sense performs to synthesise a normative system that solves the norm synthesis problem in Definition 7. That is, *norm generation* and *utility learning* (Fig. 3(2)), and *norm replication* (Fig. 3(3)). In particular, norm generation and utility learning are achieved by *runtime observation* of the agents’ activities during MAS simulations, while norm replication is subsequently performed outside simulations.

#### Generating new normative games from observation

As introduced in Sect. 3.1, sense takes the norm generation approach of iron (Sect. 2.1). It monitors agents’ interactions at regular time intervals within a simulation of the MAS for a given number of time steps. At each time step, sense tries to detect new, untracked games, and generates norms to coordinate the involved agents. Likewise iron, we assume that conflicts can be detected at runtime, and that the agents involved in a conflict are the ones that are responsible for the conflict. Moreover, we assume that a conflict in a MAS state at a given time *t* is caused by the actions that the agents performed in the previous MAS state at time $t-1$.

In order to generate norms for a given domain, sense requires the use of domain-dependent knowledge. With this aim, it relies on the following domain-dependent functions considered by iron in [17, 18] (see Sect. 2.1):

- A *conflict function* of the form $conflicts : S \rightarrow 2^{Ag}$, which returns groups of agents that are involved in a conflict in the state of the MAS at a given time.
- A *context function* of the form $context : Ag \times S \rightarrow 2^{{\mathcal {L}}}$, which returns the local context of an agent in a state of the MAS. As introduced in Sect. 3.2, this context is expressed in terms of an agent language ${\mathcal {L}}$.
- An *action function* of the form $action : Ag \times S \times S \rightarrow Ac$, which returns the action that an agent performed during the transition from a MAS state $s_t$ at a given time *t* to the subsequent MAS state $s_{t+1}$ at time $t+1$.

At a given time *t*, sense detects and keeps track of a new *m*-role game by performing the following tasks:

1. *Detecting a new conflict at time* *t*. Formally, sense obtains the conflicts of the current state of the MAS $$s_t \in S$$ at a given time *t* by invoking function $$ conflicts (s_t)$$.
2. *Retrieving the contexts at time* $t-1$ *of the* *m* *agents that are in conflict at time* *t*. This amounts to generating, for each agent $ag \in Ag$ involved in the conflict at time *t*, an expression $\varphi \in {\mathcal {L}}$ that describes the context of this agent in the MAS state $s_{t-1} \in S$ at time $t-1$. Formally, $context(ag,s_{t-1})$.
3. *Creating a new* *m*-*role game* as $G=\langle R, A, \varphi \rangle$ with *R* as the set of roles played by the agents at time $t-1$, *A* as the set of the actions available to these agents at time $t-1$, and $\varphi$ as the set of contexts of these agents at time $t-1$.

After tracking a new game *G*, sense will create *all* the norms that can be used to regulate the agents in *G*, i.e., its norm space $N_{G}$, by:

1. *Identifying the actions* that led to the conflict at time *t*. That is, retrieving the *m* actions that the *m* agents involved in the conflict performed in the transition from the MAS state $$s_{t-1}$$ at time $$t-1$$ to its subsequent state $$s_t$$ at time *t*. sense obtains the action performed by an agent $$ag \in Ag$$ in such a state transition as $$ action (ag,s_{t-1},s_t)$$.
2. *Creating alternative norms* that prohibit different roles of *G* to perform the actions that led to the conflict. Each one of these norms will prohibit agents to perform the conflicting actions whenever they perceive the context corresponding to different roles of the game.

Then, sense will create the corresponding normative game $NG=\langle G, N_{G}, H, U\rangle$, with the new game *G*, $N_{G}$ as its norm space, *H* as its tuple of history functions,^9^ and *U* as its set of empirical utility functions. Next, the norms of $N_{G}$ will be uniformly distributed among the agents in a MAS. This guarantees that the normative systems in an agent population are heterogeneous, namely the agents will play game *NG* by using different, “competing” norms.

At this point it is worth to say that sense is not guaranteed to solve a game on its first attempt, since a game may require norms that prohibit more than one action in order to coordinate the agents. For instance, if once cars are prohibited to go forward in a given game they decide to turn to one side, still colliding, then sense will synthesise further norms that prohibit both to go forward and to turn to one side, adding them to the norm space of the game and sending the new norms to the cars in the scenario.

**Fig. 8:**
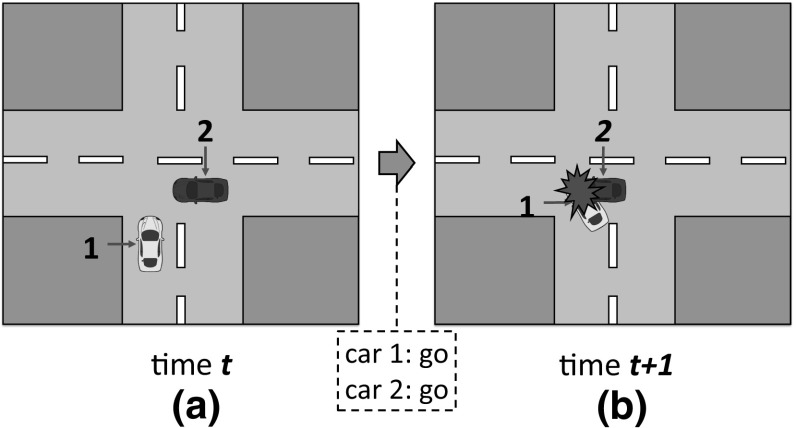
Game with two cars playing roles 1 and 2 when approaching a junction

**Fig. 9:**
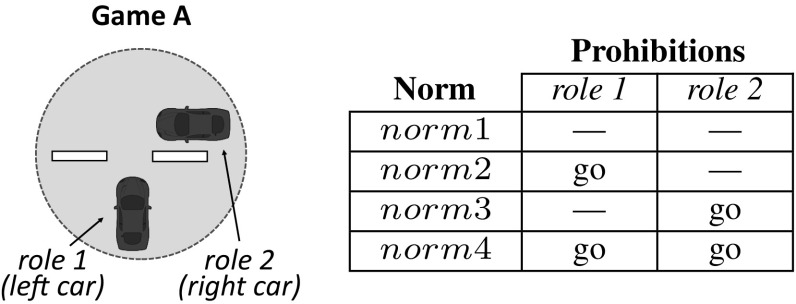
Left: game in which two cars with roles 1 and 2 need coordination when approaching a junction. Right: alternative norms to coordinate any two cars that engage in such a game

Going back to our example of $$G_A$$ and cars 1–2 (Fig. 8), sense will now create its norm space, $$N_{G_A}$$, by first identifying action *“go”* as the one performed by cars 1 and 2 during the transition from time *t* to time $$t+1$$. That is, $$ action (1, s_t, s_{t+1})=go$$ and $$ action (2, s_t, s_{t+1})=go$$. Then, it will create norms to prohibit action *“go”* to: none of the roles (*norm*1 in the Table of Fig. 9), role 1 (*norm*2), role 2 (*norm*3), and both roles (*norm*4). sense will now create and track the corresponding normative game $$NG_A=\langle G_A, N_{G_A}, H, U \rangle $$. Thereafter, it will deliver each norm to 25% of the agents.

#### Computing norms’ utilities empirically

sense continuously monitors the game play of the agents during a MAS simulation, detecting when they play each normative game, and keeping track of their rewards in the memory of the game. At the beginning of a simulation, sense *clears* the memory of each normative game detected so far in order to learn new reward histories for each game from scratch. In this way it avoids mixing reward evidences from past simulations, which may no longer apply after norms have been replicated and their frequencies have been updated. After simulation, sense will exploit this new knowledge in order to empirically compute the utility matrix of the game as follows. Let $NG=\langle G, N_{G}, H, U \rangle$ be a normative game, and $\mathbf {n}$ a combination of norms applicable to the players of *NG*. Consider the history *H* of *NG* over a time window $[t_0, t_\omega ]$. First, we propose to retrieve from *H* the rewards obtained by the agents when playing norm combination $\mathbf {n}$, which we note as $R_{\mathbf {n}}$. Then, we compute the utility obtained by player *i* as its *average reward*:

7 $$\begin{aligned} u_i(\mathbf {n}, H) = \dfrac{1}{|R_{\mathbf {n}}|} \ \sum \limits _{\mathbf {r}^t \in R_{\mathbf {n}}} \mathbf {r}^t[i] \end{aligned}$$

where $\mathbf {r}^t[i]$ is the reward obtained by player *i* in *NG* at time *t*.

**Fig. 10:**
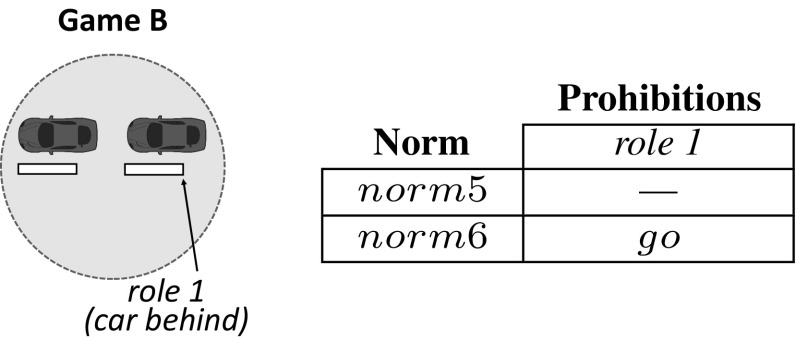
Left: game where a car behind (role 1) has to coordinate with the car in front. Right: alternative norms for this game

Back with our example, consider that after a simulation cars have played game $NG_A$ four times (Fig. 9) and game $NG_B$ one (Fig. 10). In the first game play, cars played $NG_A$ with norm combination $\mathbf {n}=\langle norm3,norm3 \rangle$ and performed joint action $\mathbf {a}=\langle go,stop \rangle$. While player 1 avoided collisions, player 2 was hit from behind by a third car that was simultaneously playing $NG_B$ behind it and did not stop because it used *norm*5. Hence, the tuple of rewards of the players of $NG_A$ at that time is $\langle 1,0 \rangle$. In the second and third game plays, cars played $NG_A$ with the same norm combination, avoiding collisions because no cars were behind them, and getting reward 1 (i.e., $\langle 1,1 \rangle$). In the fourth game play, cars played $NG_A$ with norm combination $\mathbf {n}=\langle norm1,norm1 \rangle$ and performed a joint action $\mathbf {a}=\langle go,go \rangle$, thus getting reward 0 (i.e., $\langle 0,0 \rangle$). The resulting memory of $NG_A$ is:

$$\begin{aligned} H= & {} \langle \langle \langle norm3,norm3 \rangle , \langle 1, 0 \rangle \rangle , \langle \langle norm3,norm3 \rangle , \langle 1, 1 \rangle \rangle , \\&\langle \langle norm3,norm3 \rangle , \langle 1, 1 \rangle \rangle , \langle \langle norm1,norm1 \rangle , \langle 0, 0 \rangle \rangle \rangle \end{aligned}$$

Say that we want to compute the utility of norm combination $\langle norm3,norm3 \rangle$. First, we retrieve from *H* the reward sequence of $\langle norm3,norm3 \rangle$, that is, $\langle \langle 1, 0 \rangle , \langle 1, 1 \rangle , \langle 1, 1 \rangle \rangle$. Note that $k=3$, where *k* is the length of the history of rewards for $\langle norm3,norm3 \rangle$. We will compute the utilities of this norm combination for each role as:

$$\begin{aligned} u_1(\langle norm3,norm3 \rangle , H)= & {} \dfrac{1+1+1}{3} = {\mathbf {1}} \\ u_2(\langle norm3,norm3 \rangle , H)= & {} \dfrac{0+1+1}{3} = {\mathbf {0.66}} \end{aligned}$$

#### Replicating norms

As previously introduced, norm replication is the process of computing the fitness of each norm (Definition 6 in Sect. 3.2), and then making its frequency grow proportionally to its fitness. sense computes a norm’s fitness similarly to the way a strategy’s fitness is computed in EGT (Sect. 2). Given a normative game, the fitness of a norm *n* will depend on:

1. the *utility* that an agent derives when using norm *n* to play the game by enacting different roles and by playing against other agents with possibly different norms in the game; and
2. the *probability* that the agent encounters these agents, which can be computed in terms of the frequencies of the norms applicable to these agents in the game.

Intuitively, if an agent derives a high utility once it plays a normative game against agents with a highly frequent norm, then the agent will be very likely to encounter an agent that uses that norm to play that game (and hence, to get a high fitness). Conversely, the same agent will very likely get a low fitness if it is highly likely to interact with agents against whom it always derives a low utility.

As an example, let us consider that a car repeatedly plays game $NG_A$ in Fig. 9 by using *norm*1. According to this norm, the car will never give way. This car will yield a high utility when playing against cars that have applicable *norm*4, since this norm obliges a car to always give way. This occurs when the combination of norms used in the game is either $\langle norm1, norm4 \rangle$ (with our car playing role 1), or $\langle norm4,norm1 \rangle$ (with our car playing role 2). Conversely, this car will derive a low utility when it interacts with cars that have *norm*1 (since both will go forward and collide), namely when the combination of norms used in the game is $\langle norm1,norm1 \rangle$. Now, say that the number of cars with *norm*4 doubles the number of cars with *norm*1. Then, our car will be twice as likely to play against cars that have *norm*4, and hence to obtain a higher fitness.

Given a normative game $NG=\langle G, N_{G}, H, U \rangle$, we compute the fitness of a norm *n* as the average utility $u_i$ to an agent once it uses norm *n* to play *NG*, for each role *i* and each combination of norms applicable to the players of *NG*. Formally:

8 $$\begin{aligned} f(n,NG) = \sum \limits _{i=1}^{|R|} \ \ \sum \limits _{\mathbf {n} \in N_{G}^{|R|} \ | \ \mathbf {n}_i = n} u_i(\mathbf {n}, H) \cdot p(\mathbf {n}) \end{aligned}$$

where:

- $N_{G}^{|R|}$ is the set of all norm combinations that the agents playing the game can employ;
- $\mathbf {n}$ is a norm combination and $\mathbf {n}_i = n$ is the norm employed by the agent playing role *i*;
- $u_i(\mathbf {n}, H)$ is the utility to role *i* when agents play with norm combination $\mathbf {n}$, computed based on the game’s memory *H* at a given time; and
- $p(\mathbf {n},t)$ is the joint frequency of the norms in $\mathbf {n}$ in the normative systems of the players.

We compute the joint frequency of the norms in $\mathbf {n}$ in the normative systems of the players of *NG* at a given time as:

9 $$\begin{aligned} p(\mathbf {n}) = \prod \limits _{n \in \mathbf {n}} F(n) = \frac{|\{ag \in Ag \ | \ n \in \varOmega _{ag}\}|}{|Ag|} \end{aligned}$$

where $F(n) \in [0,1]$ is the frequency of norm *n* at a given time, namely the proportion of agents whose normative systems contain *n* at that time.

**Table 2:** Example of utility matrix of *norm*1 and *norm*3 after simulation

	*norm*1	*norm*3
*norm*1	(0,0)	(1,0.45)
*norm*3	(0,0)	(1,0.66)

Once cars use *norm*1 to coordinate they go forward and collide, deriving utility 0. Once the car on the right (role 2, column player) applies *norm*3, hence stopping, cars avoid colliding. In that case, the left car (role 1, row player) derives utility 1, and the right car’s utility ranges in [0.34,0.45]

Next, those norms with higher fitness than average become more frequent, while those with fitness below average become less frequent. This will be captured by a replication equation computed as follows. If *NG* is a normative game, we update the frequency of a norm $n \in N_G$ as:

10 $$\begin{aligned} F(n) = F(n) \cdot \dfrac{f(n,NG)}{\varTheta } \end{aligned}$$

where $\varTheta$ is the average fitness of all the norms applicable in *NG*, computed as:

11 $$\begin{aligned} \varTheta = \sum _{n \in N_G} f(n,NG) \cdot F(n) \end{aligned}$$

Notice that equations 10 and 11 are the counterparts of 3-4 and 5 introduced in Sect. 2.2 to describe the replicator dynamics of EGT. Also, note that norms’ fitness will range in [0,1] since they are computed based on utilities and frequencies (which range in [0,1]).

Let us compute the norms’ fitnesses of game $NG_A$ (Fig. 9). For simplicity, let us consider that only *norm*1 and *norm*3 are available to the cars. At the outset, half of the cars have *norm*1 in their normative systems, while the rest of cars have *norm*3. Thus, it follows that the cars can employ the following norm combinations to play the game: $\langle norm1,norm1 \rangle$, $\langle norm1,norm3 \rangle$, $\langle norm3,norm1 \rangle$, and $\langle norm3,norm3 \rangle$. The joint probability of each of these combinations is 0.25 (e.g., $p(\langle norm1,norm1 \rangle ) = F(norm1) \cdot F(norm1) = 0.5 \cdot 0.5 = 0.25$). Also, let us consider that at a given time our system computes the utility matrix illustrated in Table 2 based on the memory of $NG_A$ after a MAS simulation. Then, we can compute the fitness of *norm*1 by using equation 8 as follows:

$$\begin{aligned} f(norm1,NG_A)= & {} + \ u_1(\langle norm1,norm1 \rangle , H) \cdot p(\langle norm1,norm1 \rangle ) \\&+ \ u_1(\langle norm1,norm3 \rangle , H) \cdot p(\langle norm1,norm3 \rangle ) \\&+ \ u_2( \langle norm1,norm1 \rangle , H) \cdot p(\langle norm1,norm1 \rangle ) \\&+ \ u_2(\langle norm3,norm1 \rangle , H) \cdot p(\langle norm3,norm1 \rangle )\\= & {} \ \mathbf{0 } \cdot 0.25 + \mathbf{1 } \cdot 0.25 + \mathbf{0 } \cdot 0.25 + \mathbf{0 } \cdot 0.25 =\mathbf {0.25} \end{aligned}$$

Analogously, we compute the fitness of *norm*3 as follows:

$$\begin{aligned} f(norm3,NG_A)= & {} \ u_1(\langle norm3,norm1 \rangle , H) \cdot p(\langle norm3,norm1 \rangle ) \\&+ \ u_1(\langle norm3,norm3 \rangle , H) \cdot p(\langle norm3,norm3 \rangle ) \\&+ \ u_2(\langle norm1,norm3 \rangle , H) \cdot p(\langle norm1,norm3 \rangle ) \\&+ \ u_2(\langle norm3,norm3 \rangle , H) \cdot p(\langle norm3,norm3 \rangle ) \\= & {} \ \mathbf{0 } \cdot 0.25 + \mathbf{1 } \cdot 0.25 + \mathbf 0.45 \cdot 0.25 + \mathbf{0.66 } \cdot 0.25 \simeq {\mathbf {0.528}} \end{aligned}$$

It is worth noticing that *norm*3 is more than twice as fit as *norm*1. Now, let us replicate both norms. We compute the average fitness of *norm*1 and *norm*3 using equation 11 as:

$$\begin{aligned} \varTheta= & {} f(norm1,NG_A) \cdot F(norm1) + f(norm3,NG_A) \cdot F(norm3) \\= & {} 0.25 \cdot 0.5 + {0.528} \cdot 0.5 = {\mathbf {0.389}} \end{aligned}$$

Since *norm*3’s fitness is larger than the average, its frequency in the next generation must increase, while that of *norm*1 must decrease. Specifically:

$$\begin{aligned} F(norm1)= & {} F(norm1) \cdot f(norm1,NG_A) \ / \ \varTheta \\= & {} 0.5 \cdot 0.25 \ / \ {0.389} \simeq {\mathbf {0.321}}\\ F(norm3)= & {} F(norm3) \cdot f(norm3,NG_A) \ / \ \varTheta \\= & {} 0.5 \cdot {0.528} \ / \ {0.389} \simeq {\mathbf {0.679}} \end{aligned}$$

Hence, in next generation approximately $68\%$ of agents will have *norm*3 in their normative systems, which means that *norm*3 will spread. The remaining $32\%$ of agents will adopt *norm*1, and hence the presence of *norm*1 in the agents’ normative systems will shrink.

## Empirical analysis and results

In this section we empirically evaluate our approach in a simulated traffic scenario. We explore several dimensions. Firstly, we analyse its *convergence*, showing that it manages to converge up to 100% of times to an evolutionarily stable normative system (ESNS) that fulfils the coordination needs of a given agent population. Secondly, we perform an *interdepedencies* analysis in which we analyse the effects of the interdependencies between games on the final normative systems that sense converges to. Thirdly, we test the *adaptiveness* of our approach, that is, its capability to adapt the normative systems it synthesises to the coordination needs of the population. Finally, we study the *stability* of the normative systems synthesised by our approach upon convergence. We demonstrate that, once all cars abide by an ESNS synthesised by sense, there is no alternative normative system that the agents can be tempted to switch to.

### Empirical settings

**Fig. 11:**
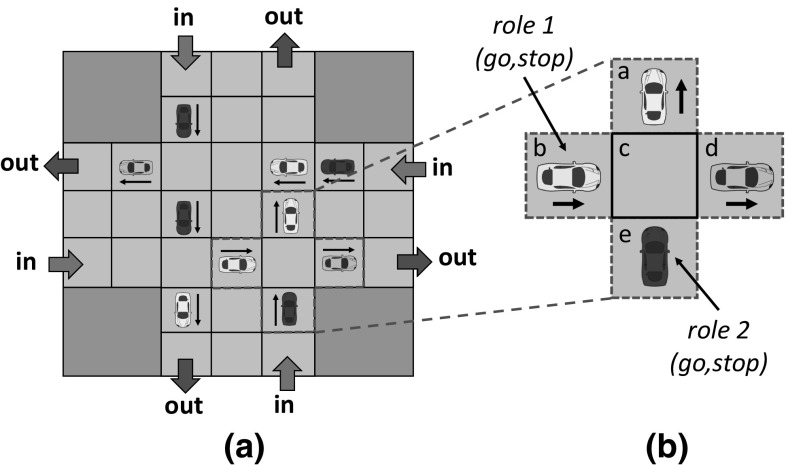
**a** Simulated traffic scenario with four entry points (labelled as in), and four exit points (labelled as out); **b** A 2-role game. Cells *a–d* describe the four positions in front of the car playing role 2 (the car at cell *e*). Cells *a, c, e* and *d* describe the four positions in front of the car playing role 1 (the car at cell *b*)

Our experiments consider a discrete simulator of a traffic scenario publicly available in [16], in which agents are cars and the coordination task is to ensure that cars reach their destinations as soon as possible without colliding. The implementation of sense used in our experiments is publicly available in [15]. Figure 11a illustrates our scenario, composed of two orthogonal roads represented by a $7 \times 7$ grid. At each time step, new cars may enter the scenario from four different entry points (labelled as “in”), and travel towards one of four exit points (labelled as “out”).

At each time step, each car performs an action out of a set of available actions *{go,acc,stop}*. Actions *“go”* and *“acc”* imply that a car moves either *one* cell or *two* cells towards its direction, respectively. Action *“stop”* implies that a car stays in its cell for one time step. Cars are *naive* and have basic driving skills, but they do not know how to coordinate. We will model a population of ***prudent drivers*** that like driving calmly and without suddenly accelerating. Thus, at a given time step, a car chooses an action to perform based on the following total order of preferences over the set of available actions:

12 $$\begin{aligned} go> stop > acc \end{aligned}$$

That is, cars perform action *“go”* by default unless they are explicitly prohibited by a norm to do so. In this case, they perform action *“stop”*, remaining in place for one time step, or action *“acc”* in case they are both prohibited to go and to stop.

Also, we consider the following reward function that numerically quantifies the personal “success” of a *car* at a given time in terms of its own goal achievement (see Definition 4 in Sect. 3.3.2):

13 $$\begin{aligned} r^t(car) = \left\{ \begin{array}{lll} 1\ \ \ &{} \hbox {if } \text {player } i ~\text {avoids collisions and can} ~\textit{go}~ \text {forward at time }t \\ 0.7\ \ \ &{} \hbox {if } \text {player } i ~\text {avoids collisions but has to}~ \textit{stop}~ \text {at time }t \\ 0.35\ \ \ &{} \hbox {if } \text {player } i~ \text {avoids collisions but has to} ~\textit{accelerate}~ \text {at time }t \\ 0\ \ \ &{} \hbox {if } \text {player } i~ \textit{collides}~ \text {at time }~t \end{array} \right. \end{aligned}$$

where $\mathbf {a}$ is a joint action performed by the players of a normative game *NG* at time *t*.

Thus, a car gets the best possible reward (reward 1) once it plays a normative game at time *t* and avoids collisions by going forward at time $t+1$ (hence not delaying). A car gets a less positive reward (reward 0.7) once it has to stop in order to not collide (which is detrimental to the goal of reaching its destination as soon as possible). When a car is required to suddenly accelerate in order to not collide, it gets half the reward it gets when stopping (0.35). Finally, a car gets the worst possible reward (reward 0) once it plays a normative game and collides, not being able to progress any more. Note that the rewards for not colliding (either by moving forward, stopping or accelerating) are significantly higher than the reward for colliding. Thus, cars will give a higher importance to avoiding collisions at the expense of travelling time. In other words, we say that the cars will be highly ***averse to colliding***.

Each car has a limited perception of the MAS and perceives the four cells immediately next to and in front of it: one cell on its left, two consecutive cells in front, and one cell on its right. For instance, in Fig. 11b the car in cell *e* can perceive cells *a, b, c* and *d*. A cell can contain either a car heading different directions, nothing, or a collision. While cars can perceive the position and direction of other cars in their contexts, they cannot assess whether they are moving (going forward or accelerating) or stopped.

We say that two cars have collided in a state if they are in the same cell at that state. Specifically, two cars collide once their trajectories coincide in time and space. For example, say that at a given time the cars in cells *b* and *e* in Fig. 11b jointly accelerate, aiming to progress two cells all at once. The trajectories of these cars will cross in cell *c*, and hence a collision between these cars will arise in this cell at the next time step.

**Fig. 12:**
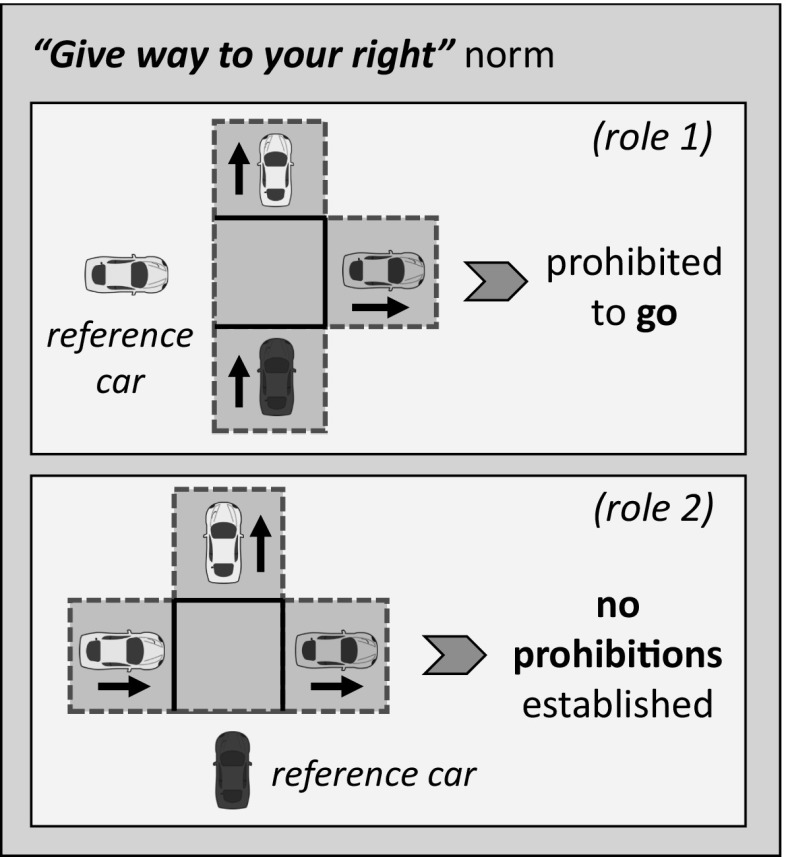
**a** Example of a *“give way to your right”* norm to regulate the game depicted in Fig. 11b. Whenever a car perceives the context of player 1 in Fig. 11b (the car in cell *b*), then it enacts role 1 in the game and hence is prohibited to *“go”*. If a car perceives the context of player 2 (the one in cell *e*), then no prohibitions are established

A game is described by means of the contexts perceived by its players. Figure 11b graphically illustrates the description of a 2-role game played by two of the cars in Fig. 11a. Two cars can avoid collisions in this game once one of them *stops* for one time step (giving way to the other car), or if it *accelerates* for one time step, anticipating to the other car and rapidly going through the junction. Conversely, if both cars go forward or accelerate at the same time, they will inevitably collide.

Norms specify the actions that cars are prohibited to perform once they enact each possible role in a game. Figure 12 graphically illustrates an example of a *“give way to your right”* norm to coordinate cars in the game depicted in Fig. 11b. Whenever a car perceives the context of role 1 in Fig. 11b (the car in cell *b*), then this car knows that it is enacting role 1 in the game and hence is prohibited to *“go”*. Conversely, a car has no established prohibitions whenever it perceives the context of player 2 (the one in cell *e* in Fig. 11b).

Each experiment consists of a set of executions of sense that start with a population of agents that have no norms to coordinate (that is, each agent $ag_j \in Ag$ has an empty normative system $\varOmega _j=\emptyset$). Simulations run in rounds of 200 time steps. In each round, cars interact in the scenario and collisions occur as the simulation goes on.^10^ sense monitors the simulation, detecting games and creating norms as detailed in Sect. 3.3.1. At the end of each simulation, sense computes norm utilities and replicates norms as detailed in Sects. 3.3.2 and 3.3.3. We consider that sense has converged to an ESNS once all the agents have adopted the same norm in each possible game, and this condition holds for 100 generations (${\mathcal {I}}=100$, see Sect. 3.1)

### Convergence analysis

We first analyse the capability of our approach to synthesise an ESNS that avoid collisions by adapting to the coordination needs of our population of prudent drivers.

**Fig. 13:**
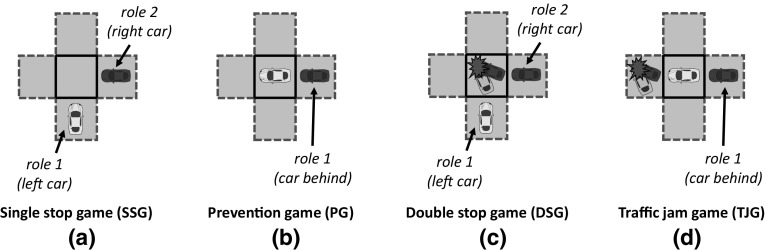
Different game types detected in our simulations: **a** a 2-role *single-stop game* (SSG), where the best strategy is that one of the cars goes forward, while the other one stops, giving way to the first car, or accelerates anticipating to it; **b** a 1-role *prevention game* (PG), in which the car playing role 1 may need to stop in order to not collide with the car in front (which might be playing a SSG); **c** a 2-role *double-stop game* (DSG), in which both cars need to stop in order to not collide because of a blocked road; **d** a 1-role *traffic jam game* (TJG), in which the car playing role 1 has always to stop in order to not collide with the car in front, which will likely stop because the road is blocked

Out of 1,000 executions of sense, the system takes an average of 54 rounds to converge. On average, sense detected 89 different games that can be grouped into the four categories illustrated in Fig. 13. The first category (label *a*), which we call *single-stop games* (SSG), stands for 2-role games in which the best strategy to avoid collisions is that one of the cars goes forward while the other car either: (i) *stops*, giving way to the first car, or (ii) *accelerates*, going through the junction before the first car does. Two examples of SSG are the game illustrated in Fig. 13a and the game depicted in Fig. 11b, which is very similar to the former one but also considers a third and a fourth car in cells *a* and *d*. In general, *any variation* in cells *a, c* and *d* of a 2-role game is considered as a different game.^11^

The second category (label *b*), which we call *prevention games* (PG), stands for 1-role games that are ***interdependent with SSGs***. In a PG, a car perceives another one in front, but not the other way around. Therefore, *only one* car (the one behind) is aware of the need for coordination of both cars. The car in front may be playing a SSG with a third car and hence may possibly stop in order to give way to it. Player 1 has no means to assess whether or not this car will stop, and thus it can choose to go forward, assuming a collision risk in case the car in front stops, or to stop for one time step in order to keep a security distance with it.

The third category (label *c*), called *double-stop games* (DSG), stands for 2-role games in which both players need to stop in order to avoid collisions because the junction is blocked. Figure 13c shows an example of DSG, in which two cars are waiting for a collision to be removed. The fourth category (label *d*), which we call traffic-jam games (TJG), stands for 1-role games that are ***interdependent with DSGs***. TJGs are similar to PGs, but in this case the road is blocked in front of the two cars and hence the car in front is very likely to stop. Thus, player 1 has no choice but stopping in order to avoid collisions. TJGs also include games in which the only player perceives a collision in the cell immediately in front of it, in which case it is also required to stop in order to avoid collisions.

It is worth noticing that SSGs and DSGs are only played *at the junction* (once the trajectories of two cars cross orthogonally), while PGs and TJGs are played *before* arriving at the junction, once cars are waiting behind other cars playing SSGs and DSGs in the junction.

Overall, sense detects 89 different games out of all executions. Thus, normative systems have 89 norms, one for each possible game. More specifically, 17 SSGs are detected, 25 PGs, 7 DSGs and 40 TJGs as a result of different variations in the empty cells in front of the players of these games in Fig. 13 (cars in different directions, a collision, or nothing). Tables 3 and 4 illustrate the possible types of norms that can be used to coordinate cars in each one of these games, along with the *percentage* of times that each type of game converged to each one of these norms. Below each percentage is the strategy adopted by the cars once they adopted each possible norm. Such a strategy stands for the actions actually performed by the cars when playing each role of a game out of the remaining actions permitted by their adopted norm. These norms specify that: *“no prohibitions”* are imposed on roles 1 and 2; “role 1 is prohibited to go” (denoted as *“role 1: prh(go)”*); “role 2 is prohibited to go” (denoted as *“role 2: prh(go)”*); and “both roles are prohibited to go”.

**Table 3:** Norms to coordinate a population of prudent drivers in interdependent SSGs and PGs, along with the percentage of times that cars converged to adopting each type of norm in these games

Norms	“no prohibitions”	“role 1: prh(go)”	“role 2: prh(go)”	“both roles: prh(go)”
SSG	0%	49%	51%	0%
*(strategy)*	(*go*, *go*)	(*stop*, *go*)	(*go*, *stop*)	(*stop*, *stop*)
PG	–	100%	–	–
*(strategy)*	(*go*)	(*stop*)	–	–

**Table 4:** Norms to coordinate a population of prudent drivers in interdependent DSGs and TJGs, along with the percentage of times that cars converged to adopting each type of norm in these games

Norms	“no prohibitions”	“role 1: prh(go)”	“role 2: prh(go)”	“both roles: prh(go)”
DSGs	0%	0%	0%	100%
*(strategy)*	(*go*, *go*)	(*stop*, *go*)	(*go*, *stop*)	(*stop*, *stop*)
TJGs	0%	100%	–	–
*(strategy)*	(*go*)	(*stop*)	–	–

On average, cars converge 100% of executions to an ESNS that avoids collisions. In SSGs (Table 3) cars adopt 49% of times norms that prohibit role 1 to go, hence adopting a *“give way to the right”* strategy. That is, they stop when playing role 1 because action *“stop”* is the next one in order of preference once action *“go”* is forbidden (see Equation 12). Since role 2 has no prohibitions, cars *go* forward when playing this role. The resulting strategy is denoted as (*stop*, *go*). The remaining 51% of times cars converge to norms that prohibit role 2 to go, hence adopting a *“give way to the left”* strategy, denoted as (*go*, *stop*). As for PGs (Table 3), 100% of executions converge to norms that prohibit to go forward and cars adopt a *“stop”* strategy.

In DSGs, cars converge 100% of times to a *“both roles are prohibited to go”* norm, and cars adopt an *“stop always”* strategy (*stop*, *stop*). Consequently, in TJGs cars converge 100% of times to a norm that prohibit them to go, also adopting a *“stop”* strategy.

**Fig. 14:**
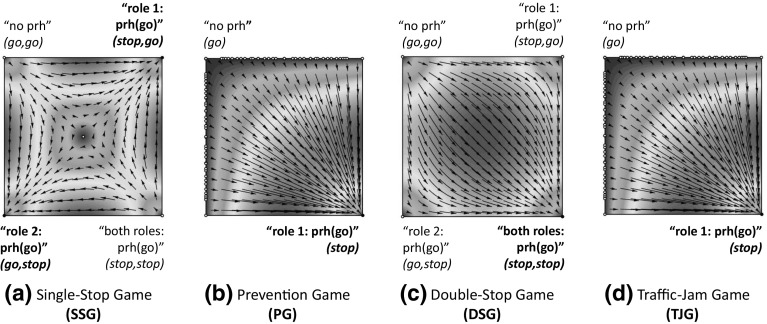
Evolutionary dynamics of norm adoption in SSGs (**a**), PGs (**b**), DSGs (**c**) and TJGs (**d**). Each square represents the possible frequency distributions of the norms in Tables 3, 4. Arrows represent the gradient of norm adoption for each norm distribution, i.e., the most likely trajectory in terms of norm adoption that a population with a given norm distribution will follow. **a** shows the dynamics of SSGs, in which any norms prescribing strategies to prohibit either role 1 or 2 to go are evolutionarily stable. **b** shows the dynamics of PGs, in which the only norm that can **always** avoid collisions is to prohibit role 1 to go. **c** shows the dynamics of DSGs, in which the only stable norm is one to prohibit both roles to go. **d** shows the dynamics of TJGs, whose dynamics are equal to those of PGs

Figure 14 shows the evolutionary dynamics of norm adoption for SSGs (a), PGs (b) DSGs (c) and TJGs (d). Each square represents the possible frequency distributions of the norms from Tables 3, 4. For instance, the top-left corner of each square represents a population in which 100% of cars adopt a *“no prohibitions established”* norm, and the middle point of the square represents a population in which the four norms are 25% frequent. Arrows represent the gradient of norm adoption for each norm distribution, i.e., the most likely trajectory in terms of norm adoption that a population with a given norm distribution will follow.

In SSGs, cars always tend to adopt norms to either prohibiting role 1 to go (*“give way to the right”*) or prohibiting role 2 to go (*“give way to the left”*). Both norms are attractor points of the norm evolution process. If the mass of cars giving way to the right is bigger than the mass of cars giving way to the left, then all agents will tend to give way to the right in order to synchronise. As for PGs, the only attractor norm is the one that prohibits role 1 to go. Regarding DSGs, cars always tend to adopt norms to prohibit both roles to go. Thus, no matter what the initial norm distribution is, as long as at least one car adopts such a norm its fitness will be higher than that of any other car, and the whole population will eventually adopt its norm. Consequently, cars tend to adopt norms to prohibit role 1 to go in TJGs.

### Interdependencies analysis

We now analyse the effects of the interdependencies between games on the convergence of sense. With this aim, we perform a second experiment with an alternative, *dynamic* population of cars that behave differently depending on their location in the scenario. When a car enters the scenario, it behaves *aggressively*, having a strong preference for quickly reaching its destination at the expense of avoiding collisions. Once cars reach the junction, they calm down and behave *more prudently*, being willing to stop if necessary. Thus, once cars engage in a PG or a TJG before reaching the junction, they choose their actions based on the following total order of preferences over the set of available actions:

14 $$\begin{aligned} go> acc > stop \end{aligned}$$

That is, cars prefer to *“go”* unless they are explicitly prohibited by a norm to do so. In that case, they prefer to accelerate rather than stopping, unless they are also prohibited to perform this action.

Once cars reach the junction and possibly play a SSG or a DSG, they behave prudently and choose their actions based on the action preference previously used in Sect. 4.2:

15 $$\begin{aligned} go> stop > acc \end{aligned}$$

Also, we consider an alternative reward function that returns higher rewards for any *car* whenever it accelerates than when it stops:

16 $$\begin{aligned} r^t(car) = \left\{ \begin{array}{lll} 1\ \ \ &{} \hbox {if } \text {player } i ~\text {avoids collisions and can} ~\textit{go}~ \text {forward at time }t \\ 0.7\ \ \ &{} \hbox {if } \text {player } i ~\text {avoids collisions and can}~ {{\varvec{accelerate}}} ~\text {at time }t \\ 0.35\ \ \ &{} \hbox {if } \text {player } i~ \text { avoids collisions but has to}~ {{\varvec{stop}}}~ \text {at time }t \\ 0\ \ \ &{} \hbox {if } \text {player } i~ \textit{csollides} ~\text {at time }~t \end{array} \right. \end{aligned}$$

We performed 1,000 executions of sense with this population. Table 5 illustrates the four types of norms that can coordinate cars in SSGs and PGs, along with: (i) the percentage of times that each type of game converged to each one of these norms; and (ii) the strategy adopted by the agents once having each norm.

Initially, one would expect that cars should adopt norms that prohibit them to go forward once enacting one of the roles in SSGs, likewise in the experiments of Sect. 4.2. However, once cars play a SSG, other cars playing PGs behind them behave aggressively, going forward and colliding with them. In other words, the players of PGs “push” the players of SSGs to adopt alternative strategies in order to avoid collisions. Then, cars converge in SSGs to norms that prohibit them both to *go* forward and to *stop* when enacting either role 1 or 2. Consequently, cars perform action *“acc”* because it is the only remaining action that is not prohibited by the norm. In other words, cars adopt an *“accelerate to anticipate to the other car”* strategy. On average, cars adopt 50% of times norms to accelerate when coming from the left (*acc*, *go*), and the remaining 50% of times they adopt norms to accelerate when coming from the right (*go*, *acc*). This allows cars to converge 100% of times to a “no prohibitions” norm in PGs that allows them to adopt a *“go”* strategy.

**Table 5:** Norms to coordinate prudent drivers in SSGs with aggressive drivers in PGs, along with the percentage of times that cars converged to adopting each type of norm in these games

Norms	“no prohibitions”	“role 1:prh(go,stop)”	“role 2: prh(go,stop)”	“both roles:prh(go,stop)”
SSGs	0%	50%	50%	0%
*(strategy)*	(*go*, *go*)	(*acc*, *go*)	(*go*, *acc*)	(*acc*, *acc*)
PGs	100%	0%	–	–
*(strategy)*	(*go*)	–	–	–

**Table 6:** Norms to coordinate prudent drivers in interdependent DSGs, along with the percentage of times that cars converged to adopting each type of norm in these games

Norms	“no prohibitions”	“role 1: prh(go)”	“role 2: prh(go)”	“both roles: prh(go)”
DSGs	0%	0%	0%	100%
*(strategy)*	(*go*, *go*)	(*stop*, *go*)	(*go*, *stop*)	(*stop*, *stop*)

**Table 7:** Norms to coordinate aggressive drivers in TJGs, along with the percentage of times that cars converged to adopting each type of norm in these games

Norms	“no prohibitions”	“role 1: prh(go,acc)”
TJGs	0%	100%
*(strategy)*	(*go*)	(*stop*)

**Fig. 15:**
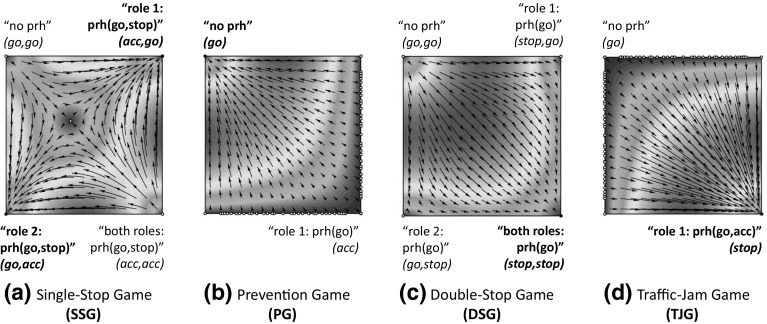
Evolutionary dynamics of norm adoption in SSGs (**a**), PGs (**b**), DSGs (**c**) and TJGs (**d**) with an alternative population of aggressive–prudent drivers. Each square represents the possible frequency distributions of the norms in Tables 5, 6 and 7. Arrows represent the gradient of norm adoption for each norm distribution, i.e., the most likely trajectory in terms of norm adoption that a population with a given norm distribution will follow. **a** shows the dynamics of SSGs, in which any norms prescribing strategies to “accelerate from the right” or “accelerate from the left” are evolutionarily stable. **b** shows the dynamics of PGs, in cars tend to adopt norms that allow them to accelerate. **c** shows the dynamics of DSGs, in which the evolutionarily stable norms are those prescribing strategy “give way always”. **d** shows the dynamics of TJGs, in which cars tend to adopt norms that prohibit them to go and accelerate, adopting a *“stop”* strategy

As for DSGs (Table 6) we observe similar convergence results to the ones illustrated in Sect. 4.2. Despite the aggressiveness of cars in TJGs, the best strategy in DSGs is still 100% of times to *“stop always”* in order to not collide with whatever is blocking the road. This provokes cars to converge 100% to norms that prohibit to *go* forward and to *accelerate* when playing a TJG (Table 7), hence adopting a *“stop”* strategy. Figure 15 shows the effects of games interdependencies on the dynamics of norms adoption. Because cars always tend to adopt a *“go”* strategy in PGs, they also tend to adopt strategies to accelerate from either the left or the right in SSGs (label *a*). The dynamics of DSGs and TJGs are similar to the ones observed in Sect. 4.2.

Notice therefore that SSGs and PGs ***cannot be resolved in isolation*** (separately), for the resulting normative system would not be evolutionarily stable. The rationale is as follows. If we disregard PGs, we can evolve norms in a SSG until the process converges to a norm that prohibits cars to “give way” to one side. Such a norm will successfully avoid collisions when cars play the SSG in isolation, for no other cars could affect the outcome of the game. Similarly, by disregarding SSGs we can evolve norms in a PG until converging to a *“no prohibitions”* norm, for the car in front would never be playing a SSG-DSG and hence would never stop. But, if we provide with these norms and put them to simultaneously play SSGs and PGs at runtime, they will not avoid collisions: cars will go forward in PGs, hitting the players of SSGs from behind. By co-evolving norms from interdependent games together, sense can synthesise a normative system that coordinates cars when playing SSGs and PGs both in isolation and simultaneously.

### Adaptiveness analysis

Next, we analyse the capability of sense to adapt norm synthesis to the coordination needs of the population to regulate. With this aim, we run simulations with *prudent drivers* that have different degrees of *aversion to colliding*. Cars choose their actions at a given time by employing the action total order depicted in Equation 15 from Sect. 4.3.

**Table 8:** Reward functions to model populations with different degrees of collision aversion. The lower rewards (e.g., $r^t_0, r^t_1$) represent populations with lower aversion to colliding. The higher rewards (e.g., $r^t_9, r^t_{10}$) represent populations with higher aversion to colliding

Outcome	$r^t_0$	$r^t_1$	$r^t_2$	$r^t_3$	$r^t_4$	$r^t_5$	$r^t_6$	$r^t_7$	$r^t_8$	$r^t_9$	$r^t_{10}$
Goes and avoids collisions	1	1	1	1	1	1	1	1	1	1	1
Stops and avoids collisions	0	0.1	0.2	0.3	0.4	0.5	0.6	0.7	0.8	0.9	1
Accelerates and avoids collisions	0	0.05	0.1	0.15	0.2	0.25	0.3	0.35	0.4	0.45	0.5
Collides	0	0	0	0	0	0	0	0	0	0	0

**Fig. 16:**
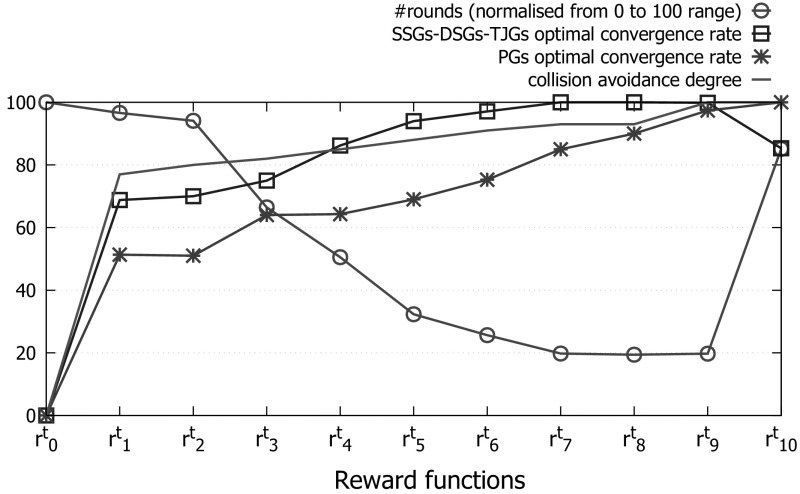
Averaged results of 1000 simulations for each collision aversion degree. The *x*-axis represents different reward functions modelling different collision aversion degrees. For instance, $r^t_0$ represents a population with null collision aversion, and $r^t_{10}$ represents a population with total collision aversion. The *y*-axis shows: (1) the number of rounds the simulations required to converge (normalised in the 0–100 range, where 100 represents the maximum number of rounds required in all simulations, i.e., 278); (2) the frequency with which cars converge optimally in SSGs, DSGs and TJGs; (3) the frequency with which cars converge optimally in PGs; and (4) the collision avoidance rate during the last round of the simulation (once agents had converged to an ESNS)

We model these populations by considering a collection of reward functions depicted in Table 8. Each function returns 0 once a car collides at time *t*, and 1 once a car goes forward without colliding at time *t*. These functions differ in the reward obtained by the cars once they *stop* or *accelerate* to avoid collisions, which balances their “hurry” to get to their destinations with their willingness to avoid collisions. In particular, the reward for accelerating is always half of the reward for stopping. This is to preserve cars’ preferences for stopping instead of accelerating. If these rewards are low (e.g., $r^t_0$), then cars prefer not to stop in order to not delay, even if it implies a collision risk. Then, we say that cars have a *low* collision aversion degree. As these rewards increase, cars have a lower aversion to stopping, which can be interpreted as a higher aversion to colliding. We run 1,000 simulations for each reward function. Figure 16 shows averaged results of all simulations. The *x*-axis depicts the different empirical reward functions (collision aversion degrees), and the *y*-axis shows:

- the number of rounds that sense requires to converge, normalised from 0 to 100 range, (where 100 represents the average maximum number of rounds required to converge out of all simulations).
- the average frequency with which cars optimally converge in the games that achieved 100% successful convergence in Sect. 4.2: SSGs, DSGs and TJGs. That is, the frequency with which they converge to a norm like “give way to the right” or a norm like “give way to the left” in SSGs, and to a norm like “always stop” in DSGs and TJGs.
- the average frequency with which cars optimally converge in PGs. That is, the frequency with which they converge to a norm that prohibits to go. In fact, this norm is the only one that allows to avoid 100% of collisions in PGs.
- the collision avoidance rate during the last round of the simulation (once the simulation has converged and the cars have adopted an ESNS).

With null collision aversion ($r^t_0$), simulations take the highest number of rounds to converge, (278 rounds, normalised as 100 in the plot) . This happens because the rewards for colliding and for stopping are equal, and hence the fitness of the norms that cause collisions (those establishing no prohibitions) and those that prohibit to go and avoid collisions are similar. Consequently, cars take a long time to decide which norm to adopt. Upon convergence, cars adopt ESNSs containing norms that establish no prohibitions, hence avoiding 0% of collisions.

As the collision aversion increases, simulations take less number of rounds to converge and cars adopt norms that prohibit to go more frequently. For low collision aversions ($r^t_1$ to $r^t_3$), simulations still take a high number of rounds (269, 262 and 185 rounds, respectively, which are normalised as 96, 94 and 66.5), but cars adopt ESNSs that avoid up to 82% of collisions. Specifically, cars converge optimally up to 72% of times in SSGs, DSGs and TJGs, and up to 64% of times in PGs. The reason that this frequency is slightly lower in PGs is because in these type of games cars do not always collide once they choose to go forward. Hence, cars occasionally converge to norms establishing no prohibitions, which cannot fully avoid collisions. For middle and high collision aversion degrees ($r^t_4$ to $r^t_9$), the number of rounds necessary to converge decreases significantly. The best results are given by functions $r^t_7$ and $r^t_8$, with which convergence is achieved on an average of up to 54 rounds (normalised as 19 in the plot), and cars optimally converge in the 100% of SSGs, DSGs and TJGs – yet they optimally converge up to 91% in PGs, hence avoiding up to 93% of collisions.

With total collision aversion ($r^t_{10}$), the number of rounds necessary to converge increases again (up to 237, normalised as 85). This happens because the reward for stopping and not colliding, and the reward for going forward and not colliding are equal. Hence, the fitness of all the norms that avoid collisions in SSGs (either by prohibiting one role or both roles to go) are similar. In consequence, cars need extra time to decide which norm to adopt. Upon convergence, cars adopt ESNSs containing only norms that prohibit *both roles to go* in SSGs and DSGs, hence converging optimally for DSGs, but not for SSGs. As a result, cars remain stopped indefinitely and 100% of collisions are avoided. It turns out that cars are so afraid of colliding that they do not mind to stay indefinitely in place in order to avoid collisions.

### Stability analysis

Finally, we analyse the stability of the normative systems synthesised by our approach upon convergence. With this aim, we perform 100 different executions of sense that consider a population of 100 agents of which the 100% abide by an ESNS of those synthesised in the experiment of Sect. 4.2, which we will call $\varOmega ^*$. Each execution of sense lasts 400 rounds, i.e., 400 iterations of the evolutionary process illustrated in Fig. 3, composed of MAS simulation (Fig. 3(2)) and norm replication (Fig. 3(3, 4)). Each MAS simulation lasts 200 time steps, during which cars interact in the junction, playing different games and coordinating by means of their ESNS. In each round, during norm replication, sense randomly chooses a 10% of agents (i.e., 10 agents) to be mutated. For each one of these agents, sense randomly chooses a 10% of norms from its normative system (8.9 on average, since each ESNS contains 89 norms on average), and replaces them with random norms from the norm spaces of their games. Thus, on average, in each round sense mutates an average of 89 norms in the agents’ normative systems.

In each round, the mutant agents introduced by sense and the majority of agents adopting an ESNS will play against each other, evaluating how their norms perform, and adopting the norms that perform better during norm replication. As considered in the literature in EGT [32], we consider that our normative system is an ESNS if none of its norms can be invaded by any alternative norm. That is, if in every single round, the fitness of the mutant norms is lower than the fitness of the norms in $\varOmega ^*$ (and hence, the invader norms cannot grow in frequency). Thus, if our normative system is an ESNS, every mutant agent will end up adopting it after a certain number of rounds.

**Fig. 17:**
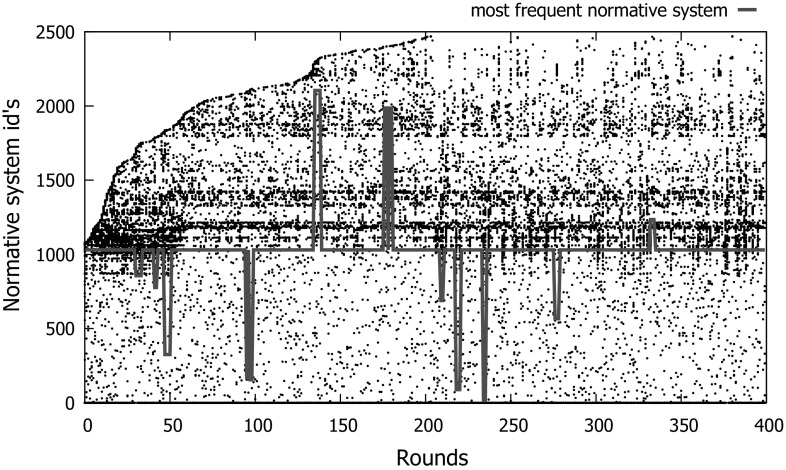
Competition of an ESNS $\varOmega ^*$ (represented with id 1000) against mutant normative systems in one simulation. The *x*-axis illustrates the different rounds of the simulation. The *y*-axis illustrates the id’s of the different normative systems generated during the simulation. Black dots represent the creation of mutant normative systems with a certain id at a given round. The red line illustrates the id of the most frequent normative system

In 100% of sense’s executions, cars ultimately adopted normative system $\varOmega ^*$. Figure 17 illustrates the dynamics of one of these simulations. The *x*-axis shows the different rounds of the simulation, and the *y*-axis depicts the id’s of the normative systems created over time. Black dots represent mutant normative systems created in each round (with which $\varOmega ^*$ has to compete), and the red line indicates the id of the most frequent normative system. For the sake of clarity, we represent $\varOmega ^*$ as the normative system with id 1,000. After 200 rounds, the simulation created 2,500 different mutant normative systems. Upon round 400, normative system $\varOmega ^*$ remained stable most of the time. In punctual rounds, sense generated a high number of mutant normative systems, making the frequency of $\varOmega ^*$ to go below stability. This happened because at certain rounds there were more mutant agents than agents with $\varOmega ^*$. But, after a few rounds, $\varOmega ^*$ replicated and mutant agents ended up adopting $\varOmega ^*$, thus becoming again the most frequent normative system. Upon round 400, the cars converged to adopting $\varOmega ^*$, thus demonstrating that $\varOmega ^*$ is a best choice for the agents.

Finally, let us detail *why* $\varOmega ^*$ remains stable over time with an example. Consider the game of our running example (Figure 13a, pp. 22) in which two cars approach each other in a junction. Say that $\varOmega ^*$, which is initially adopted by all the agents, contains a *“give way to your right”* norm, i.e., prohibited to go when enacting role 1, and allowed to go when enacting role 2. Destabilising this norm would require, at least, a 50% of mutant agents that adopt an alternative *“give way to the left”* norm. Intuitively, even if there would be a 49% of mutant agents that give way to the left, cars would still be more likely to encounter other cars that give way to the right (51% likely). Therefore, giving way to the right would still be the best choice for the agents, and hence would remain stable. However, should these probabilities be equal (once a 50% of cars are mutant), then the agents could end up adopting either of these norms. In our scenario, this applies to every Single-Stop Game (Fig. 13a) and Double-Stop Game (Fig. 13a). Since these conditions never occur during our stability experiments, $\varOmega ^*$ remains stable until the end.

## Related work

Broadly speaking, research on norm synthesis can be classified into two main strands of work: *off-line design*, and *on-line synthesis*. Pioneered by Shoham and Tennenholtz [30], off-line design aims at designing the norms that will coordinate the agents before they start to do their work [8, 11, 30, 35]. Alternatively, on-line synthesis studies how norms can come to exist while the agents interact at runtime. Most on-line approaches focus on investigating how norms *emerge* from agent societies through an iterated process whereby agents tend to adopt best-performing strategies [2, 3, 10, 29, 36, 37]. For an extensive survey about several works on norm emergence, the reader may refer to the work in [23].

Alternatively, recent work by Morales et al. approached on-line synthesis by employing designated agents that observe agents’ interactions at runtime and generate norms aimed at resolving conflict situations [18, 19]. Later on, Mashayekhi et al. [14] extended this work by proposing a hybrid mechanism in which norms are synthesised by designated agents, and the agent society iteratively selects and spread best-performing norms, ultimately adopting most successful ones.

The closest approach to our work in the literature is that of norm emergence, and particularly the study of norm evolution and stability. One of the pioneer works on this approach is the one by Axelrod [3, 4], which studies how norms evolve and emerge as stable patterns of behaviour of agent societies. Axelrod considers a game-theoretic setting in which agents repeatedly play a single two-player game by employing different strategies. The strategies that allow the agents to achieve better personal results prosper and spread. A (stable) norm is said to have emerged once a majority of agents abide by the same strategy that is sustained over time.

Subsequently, many researchers have studied norm emergence by employing the game theoretic approach. In [27], Rajiv Sethi extended the work of Axelrod and studied how social norms of vengeance and cooperation emerge within agent societies. With this aim, Sethi incorporated the solution concept of evolutionarily stable strategy (ESS) and the principle of replicator dynamics from evolutionary game theory (EGT) [32]. Again, this work considers that agents play a single two-player game, and hence one norm can be synthesised. Shoham and Tennenholtz [31] introduced a framework for the emergence of social conventions as points of (Nash) equilibria in *stochastic* settings. They introduced a natural strategy-selection rule whereby the agents eventually converge to rationally acceptable social conventions.

Later, Sen and Airiau proposed in [26] a *social learning* model whereby agents can learn their policies and norms can emerge over repeated interactions between the agents in two-player games. Many works have considered this model to study further criteria that affect to the emergence of norms. Of these, the closest to our work is perhaps the one by Sugawara et. al. [33, 34], in which conflict situations are characterised as Markov games, and a model is introduced to evolve norms that successfully coordinate the agents in these games.

More recent work (such as that by Santos et al. [21]) studies how cooperation norms can emerge once the agents can explore alternative strategies, i.e. they have arbitrary exploration rates. They show that cooperation emergence depends on both the exploration rate of the agents and the underlying norms at work. Similarly, Soham et al. [9] introduce an EGT-based model to study how norms change in agent societies in terms of the *need for coordination* and the agents’ exploration rate. They show that societies with high needs for coordination tend to lower exploration rates and higher norm compliance, while societies with lower coordination needs lead to higher exploration rates. Also, Lorini et al. [13] introduce a model for the evolution and emergence of fairness norms in relation to the degree of sensitivity (internalisation) of the agents to these norms. They show that, in the long term, the higher the sensitivity of the agents to norms, the more beneficial for the social welfare.

From Axelrod [3, 4] to Lorini [13], our approach is different to all the aforementioned works for several reasons. Most previous works consider that the agents play a *single* game whose payoffs are known beforehand. Unlike them, our framework considers a setting in which agents can play multiple, interdependent games whose outcomes may depend on each other. Our framework performs *runtime detection* of interdependent games, and automatically creates norms whose coordination utility is computed based on the rewards to the agents once they repeatedly play each game over time. To the best of our knowledge, our framework is the first one in introducing the analytical concept of evolutionarily stable normative system (ESNS) as a set of norms that, together, successfully coordinate the agents in multiple interdependent games.

## Conclusions and future work

In this work we introduced sense, a framework for the off-line synthesis of evolutionarily stable normative systems (ESNS), whose compliance forms a rational choice for the agents. sense synthesises sets of *codependent norms* that, together, successfully coordinate the agents in multiple, interdependent coordination situations that cannot be easily modelled and resolved separately beforehand. ESNSs are synthesised by carrying out a natural selection process inspired in evolutionary game theory (EGT) whereby the agents tend to adopt the norms that are more successful to coordinate them in strategic situations.

sense assumes no previous knowledge about the potential situations in which agents may need coordination, neither about their outcomes. Instead, it learns these by running MAS simulations, detecting situations that lead to undesirable outcomes, and modelling them as interdependent *one-shot games*. sense automatically synthesises norms for each game, and makes norms compete with each other in repeated game plays, iteratively learning their utilities in an empirical manner. Norms that are more useful to coordinate the agents in games prosper and spread, and are ultimately adopted by the agents. The outputs of such an evolutionary process are normative systems whose norms’ coordination utilities are co-dependent. Together, these norms are evolutionarily stable and effectively coordinate the agents in a variety of interdependent situations. Once the agents are provided with these norms, no agent can benefit from either violating them or switching to alternative ones.

We provided evidence of the quality and the relevance of our approach through an empirical evaluation in a simulated traffic scenario. We showed that our framework converges 100% of times to ESNSs that satisfy the coordination needs of car populations, avoiding collisions in a numerous (up to 89) interdependent traffic situations. We showed that the ESNSs synthesised by sense can only be synthesised by co-evolving norms from interdependent games together. Otherwise, the resulting normative systems might be ineffective and unstable. We illustrated the capability of our approach to adapt norm synthesis to the preferences of the agent population, showing that different types of normative systems can be synthesised as one considers agent populations with different preferences and behaviours.

As future work, there are multiple opportunities for research. First, we plan to enhance sense to synthesise *essential norms* [36]. sense’s model in this paper is described in terms of the individual goals of the agents, and hence, sense is capable of synthesising norms that are evolutionarily stable once they are useful for the agents to satisfy their own goals. Nevertheless, sense cannot be employed to synthesise essential norms that allow to achieve a global, system-level goal established by a system designer that might be unaligned with the agents’ goals. With this aim, we plan to add a *sanction mechanism* in sense to synthesise normative systems that achieve some system-level goals, while being evolutionarily stable.

Second, and more importantly, we plan to improve sense to synthesise ESNSs *without the need for simulation*. In this improved version of sense, a system designer will only need to provide domain information in the form of small sets of two-player games, along with their potential interdependencies represented as relationships in a games network. Based on this information, sense will carry out a co-evolutionary process that mathematically simulates the co-evolution of agents’ strategies together with norms. For instance, in the traffic scenario considered in this paper, sense could synthesise sets of essential norms that are evolutionarily stable by only considering a few (up to five) two-player games describing basic car interactions (e.g., two cars in a junction, two cars doing a queue, and so on) along with their interdependency relationships.

## Discussion

In what follows we provide guidelines about potential uses of sense. First, we describe sense’s limiting assumptions and how sense could be employed once certain conditions hold. Then, we detail how some of these assumptions could be lifted in order to apply sense to a wider variety of problems.

In order to apply sense, a scenario, besides the traffic scenario described in this paper, must satisfy the following requirements:

- *Agents must be rational*. sense assumes that agents always choose the strategies (norms) that allow them to maximise their individual payoffs (fitnesses). The assumption of rationality is a common and sensible assumption in norm synthesis research that has allowed to use ideas from game theory and evolutionary game theory to study how autonomous agents might interact and, in particular, how agents could reach successful conventions [1, 3, 22, 24–26, 31, 37].
- *Agents’ interactions can be simulated*. sense employs simulation of a MAS in order to learn its potential games along with the co-dependent utilities of their norms. Therefore, the MAS at hand should be capable of being simulated.
- *Agents’ preferences can be modelled at design time*. sense assumes some domain information that must be provided by a system designer together with the MAS simulator, such as the agents’ action preferences and rewards. For example, in Sect. 4 sense considers a reward function (Eq. 16 in page 26) that returns the individual reward of a car once it collides in some situation, once it is able to progress safely, and so on.
- *MAS conflicts must be identifiable*. Since sense’s game detection is based on the detection of conflicts (e.g., car collisions), it must be able to identify once a group of agents are engaged in a conflict.
- *Agents’ actions consequences arise immediately*. sense’s *one-shot* games detection is based on the assumption that once a conflict arises at a given time *t*, the cause of the conflict can be identified from the actions of the agents at the previous time step, $t-1$.
- *Agents’ preferences and behaviours do not change over time*. sense is intended for off-line norms design, and hence, it synthesises normative systems that are evolutionarily stable whenever agents’ preferences and actions do not change over time. However, should these conditions change at runtime, an ESNS may not be stable any more.

When the conditions above hold, sense can be successfully used to perform off-line synthesis of ESNSs even when the potential conflict situations of the MAS at hand cannot be identified beforehand. In this sense, sense stands for a novel contribution and a valuable tool for system designers. By simulating agents’ interactions (such as cars’ driving skills in a road) and providing basic domain information (such as identifying when cars collide, and assessing cars’ individual rewards after interacting), sense can automatically create ESNSs that will achieve effective and stable coordination in a MAS. To the best of our knowledge, there is no previous work in the literature in norms research that addresses the synthesis of normative systems for multiple interdependent coordination situations.

Additionally, by applying some changes, sense might be adapted to perform *on-line norm synthesis* in a decentralised manner. That is, sense might be employed by individual agents during real MAS executions in order to make on-line predictions about which norms might be evolutionarily stable. Potentially, this could allow agents to achieve fast convergence of ESNSs at runtime – instead of going through an iterative emergence process until converging to some stable convention, the agents could predict which norms are stable in advance in order to immediately adopting them. However, further work would be required to investigate this on-line strand of research and to prove its feasibility and performance.

**Fig. 18:**
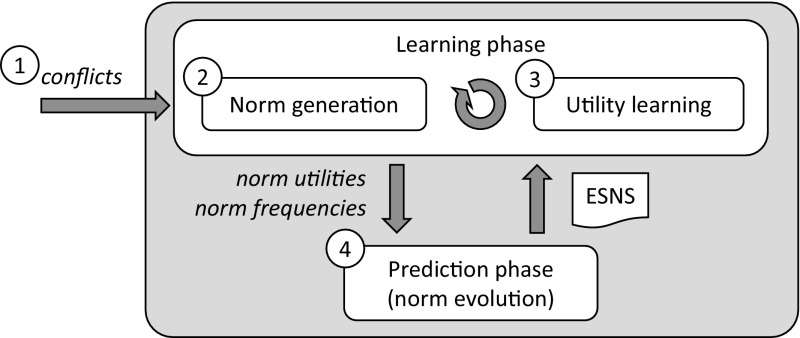
Graphical representation of how sense could be employed by individual agents to make on-line predictions about which norms might be evolutionarily stable. (1) an agent continuously interacts in a MAS, detecting conflicts; (2) for each detected conflict, the agent creates a new game and its corresponding norms in order to avoid the conflict in the future; (3) over a series of game plays, the agent accumulates evidence about the empirical utility of each norm of each game; (4) once the agent accumulates enough evidence about the utility of each norm, it performs norm evolution, which outputs an ESNS to be adopted by the agent

In order to synthesise ESNSs on-line, each agent should be embedded with a modified version of sense illustrated in Fig. 18. Each agent should proceed by continuously interacting in the MAS, detecting when it engages in conflicts (Fig. 18(1)). Based on detected conflicts, the agent could continuously *learn* (keep track of) the potential games it can play, and creating different norms to coordinate in each possible game (norm generation, Fig. 18(2)). Game detection and norm generation should be performed as illustrated in Sect. 3.3.1. Over time, the agent would play different games by enacting different roles and by playing against different agents. Unlike the original off-line version of sense, in this on-line approach the agent would always have available *all the norms* of the norm space of a game once it plays the game. In each game play, the agent should choose one norm to apply in the game out of the norm space of the game according to its frequency (probability). Thus, if a norm is highly frequent, the agent would be very likely to apply it every time it plays the game. This would allow the agent to accumulate evidence about the rewards it obtains by applying different norms in the game in order to compute the utility of each norm as described in Sect. 3.3.2 (utility learning, Fig. 18(3)). Once the agent would have enough evidence about the utility of each norm, it could internally simulate norm evolution by performing multiple norm replication steps as described in Sect. 3.3.3 (Fig. 18(4)). The output of norm evolution would be a prediction about the set of norms that might be evolutionarily stable given the current MAS conditions, i.e., an ESNS. Then, the agent should just immediately adopt these norms in order to try to achieve stable coordination. If the adopted norms were not stable, then the agent could keep on accumulating evidence about norms’ utilities and carrying out norm evolution again in order to make more accurate predictions.

Notice though that applying sense on-line requires to make some assumptions about the agents’ capabilities. Particularly, agents need to be endowed with capabilities to (1) detect conflicts; (2) assess the contexts and observe the actions of other agents in games; (3) explicitly create games along with their norms; (4) empirically evaluate norms’ utilities; and to (5) replicate norms’ probabilities.

Finally, notice that by employing sense in an on-line manner the agents could adapt their normative systems to the changing conditions of the MAS at runtime. That is, once the agents have adopted an ESNS, should the MAS’ conditions change (such as the agents’ rewards, or the type of conflicts), the agents could gather new evidence in order to evolve norms again, thus predicting new ESNSs that adapt to the new system conditions.

## Footnotes

1 iron also considers the effects of the agents not complying with norms in order to assess whether they are necessary. However, such a necessity is not relevant for the purposes of this paper.

2 Action *“go”* may include turns.

3 This function has to be characterised for each particular domain in which iron is to be employed.

4 With this aim, iron needs to monitor the MAS by means of a window of at least two time steps (that is, the current MAS state at time *t* and the previous MAS state at time $t-1$).

5 Provided that the disturbance is not too large. For example, a small number of mutant strategists joins the scenario, and after some time they are “eliminated” by dominant strategists.

6 This value must be specified by a system designer.

7 sense simulates 100% norm compliance in order to be able to evaluate norms’ utilities once the agents comply with them. However, 100% compliance is not actually required in real-time MAS execution. Once the agents are provided with an ESNS, this normative system establishes a convention for all the agents in each possible game. Such a convention has the property that no agent could benefit from violating it or from adopting different norms, and hence, agents would voluntarily comply with norms.

8 In principle, it is not possible to assume the composition of $\mathbf {a}$, i.e., the actions that these agents will perform. However, we assume that the actions in $\mathbf {a}$ *comply with* the prohibitions established by their respective norms in $\mathbf {n}$.

9 Initially, each history function $h_j \in H$ will return an empty sequence of rewards. Consequently, each empirical utility function $u_i \in U$ will return an undefined value.

10 Whenever two or more cars collide, they remain in the scenario for 5 time steps until they are removed. With this delay we aim to simulate the time that the emergency services require to remove collided cars.

11 This is because sense assumes no previous knowledge about the potential games of the MAS, and hence it cannot assume that two similar situations will in fact correspond to the same game.
